# Staphylococcal accessory regulator SarA-mediated modulation of autolysis and surface charge enables *Staphylococcus aureus* to evade vancomycin killing

**DOI:** 10.1128/msystems.01630-25

**Published:** 2026-02-09

**Authors:** Yujie Li, Shihui Yuan, Ping Yan, Shupei Zhai, Zhien He, Huimin Su, Zhongliang Zhu, Qingze He, Weifeng Xu, Baolin Sun

**Affiliations:** 1 Anhui Province Key Laboratory of Pollution Damage and Biological Control for Huaihe River Basin, School of Biological and Food Engineering, Fuyang Normal University 118409, Fuyang, China; 2 Anhui Province Key Laboratory of Embryo Development and Reproduction Regulation, School of Biological and Food Engineering, Fuyang Normal University 118409, Fuyang, China; 3 Anhui Province Key Laboratory of Environmental Hormone and Reproduction, School of Biological and Food Engineering, Fuyang Normal University 118409, Fuyang, China; 4 State Key Laboratory of Immune Response and Immunotherapy, School of Basic Medical Sciences, Division of Life Sciences and Medicine, University of Science and Technology of China 12652, Hefei, China; 5 Department of Oncology, The First Affiliated Hospital of USTC, Division of Life Sciences and Medicine, University of Science and Technology of China 12652, Hefei, Anhui, China; 6 Huaibei Agricultural Products Quality and Safety Testing Center, Huaibei, Anhui, China; 7 State Key Laboratory for Diagnosis and Treatment of Infectious Diseases, National Clinical Research Center for Infectious Diseases, Collaborative Innovation Center for Diagnosis and Treatment of Infectious Diseases, the First Affiliated Hospital, Zhejiang University School of Medicine 26441, Hangzhou, Zhejiang, China; 8 Division of Life Sciences and Medicine, University of Science and Technology of China 12652, Hefei, Anhui, China; Purdue University, West Lafayette, Indiana, USA

**Keywords:** *Staphylococcus aureus*, staphylococcal accessory regulator SarA, autolysis, bacterial surface charge, vancomycin resistance

## Abstract

*Staphylococcus aureus* is a major source of community and nosocomial infections. Due to the extensive application of antibiotics, *S. aureus* has developed resistance to antibiotics, especially vancomycin, making clinical treatment challenging. Staphylococcal accessory regulator A (SarA) modulates *S. aureus* virulence by regulating the principal virulence factors. However, its role in vancomycin resistance remains largely unknown. Herein, we found that SarA not only reduces the susceptibility of *S. aureus* to vancomycin by directly inhibiting the expression of autolysis-related genes, but also enhances resistance to vancomycin by negatively regulating the transcription of an ATP-binding cassette (ABC) transporter, ABC-like, thereby altering the bacterial surface charge and reducing vancomycin’s binding efficiency to the cell wall. Moreover, the regulation of antibiotic resistance by SarA is strain-dependent. Our study uncovers the roles of SarA in regulating vancomycin resistance, providing potential targets and ideas for the prevention and control of vancomycin-intermediate *S. aureus* infections.

**IMPORTANCE:**

*Staphylococcus aureus* poses a major threat to public health due to its increasing resistance to vancomycin, a last-line antibiotic. This study reveals that Staphylococcal accessory regulator A regulates vancomycin resistance in *S. aureus* by suppressing genes related to autolysis and negatively regulating an ATP-binding cassette (ABC) transporter (ABC-like). This regulation of the transporter reduces the bacterial surface charge, impairing the ability of vancomycin to bind to the cell wall. These findings suggest a novel mechanism of antibiotic resistance in *S. aureus* and identify potential targets for combating vancomycin-intermediate *S. aureus* infections.

## INTRODUCTION

*Staphylococcus aureus* is a major human pathogen that causes widespread infections, ranging from moderate-to-severe skin infections to fatal pneumonia and even sepsis (1–4). Due to the extensive use of antibiotics, *S. aureus* has developed resistance to multiple antibiotics, especially the cell wall-targeting antibiotic vancomycin, which has been considered the last-resort drug to treat serious infections caused by *S. aureus*, thereby further challenging clinical treatment (5–7). Therefore, it is of significant clinical relevance to investigate the mechanism of resistance of *S. aureus* to vancomycin.

Staphylococcal accessory regulator A (SarA), a prototypical member of the SarA protein family in *S. aureus*, is involved in the regulation of *S. aureus* biofilm formation and pathogenesis (8–11). The *sarA* locus is composed of three overlapping transcripts expressed from three distinct promoters, P1, P2, and P3, each encoding a major 372-bp *sarA* ORF that yields a 14.7-kDa SarA protein (12). SarA is the first member of the SarA protein family, which was found to play a crucial role in the regulation of virulence genes (12). SarA can positively and negatively regulate the expression of targets; for instance, the *sarA* locus has been shown to upregulate the synthesis of selected extracellular and cell wall-associated proteins while down-modulating the expression of the genes of protein A and protease (12). SarA can directly regulate the expression of target genes by binding to the promoters, including those of *agr*, *fnbA*, and TSST-1, indicating that the regulation can occur both in *agr*-dependent and *agr*-independent manners (8, 12). Actually, since *agr* is a well-identified regulatory system, the fact that SarA can upregulate the *agr* expression is one of the strong pieces of evidence of its role as a global regulatory factor (8, 13). SarA cannot only regulate the expression of downstream genes but also upregulate self-expression by binding to its promoter (14). In addition, *sarA* expression is modulated by other regulatory factors such as SigB and SarR (8, 15, 16).

In addition to regulating virulence, SarA can contribute to the resistance of *S. aureus* to vancomycin via cysteine phosphorylation mediated by the eukaryotic-like kinase-phosphatase pair Stk1-Stp1 (17). Past studies have demonstrated that, compared with vancomycin-susceptible *S. aureus* strains, vancomycin-intermediate *S. aureus* (VISA) strains often exhibit a phenotype of cell wall thickening and reduced autolysis activity (18, 19). Correspondingly, the expression of genes related to cell wall synthesis was upregulated, while the expression of genes related to autolysis was downregulated (18, 19). It has also been reported that SarA is involved in reducing the production of autolysins responsible for cell wall recycling (20). Nevertheless, the regulatory mechanisms involving SarA in the antibiotic resistance of *S. aureus* remain far from being fully elucidated.

ATP-binding cassette (ABC) transporters are the largest and most diverse protein superfamilies that couple ATP binding, hydrolysis, and phosphate release to deliver diverse substrates such as vitamins, steroids, lipids, ions, polysaccharides, and xenobiotics across membranes (21–23). ABC transporters usually consist of two highly conserved nucleotide-binding domains (NBDs) and two variable transmembrane domains (TMDs); these four domains are often distinct subunits or are fused into homo- or heterodimerizing half-transporters composed of one NBD and one TMD in bacteria and archaea (22, 24). ABC transporters play vital roles in various physiological processes, including the uptake of nutrients, secretion of signaling molecules and toxins, multidrug resistance, and bacterial virulence (25, 26). Shea et al. demonstrated that the ABC transporters function as virulence factors in uropathogenic *Escherichia coli* (26). For multidrug resistance, ABC transporters can detoxify drugs by transporting them outside of cells or into specialized compartments across membranes, which is part of the resistance and detoxification mechanisms (27). In addition, unlike traditional ABC transporters, ABC-F proteins are not membrane-bound, but they can also be involved in the regulation of antibiotic (e.g., macrolides, lincosamides, streptogramins, and pleuromutilins) resistance (28). However, whether the ABC transporters participate in the regulation of vancomycin resistance in *S. aureus* has not been systematically analyzed to date.

In this study, we demonstrated that SarA could regulate the resistance of *S. aureus* to vancomycin by regulating the expression of autolysis-related genes. It was also found that the regulation of the ABC transporter by SarA contributed to vancomycin resistance: SarA can regulate the expression of the ABC-like transporter to alter the surface net charge of *S. aureus*, thereby modulating its affinity for vancomycin and ultimately reducing its sensitivity to the antibiotic. This study provides new targets for the treatment of VISA infections as well as a theoretical reference for the treatment of other bacterial infections, which can be of great research significance.

## RESULTS

### SarA contributes to vancomycin resistance of *S. aureus* XN108

**Fig 1:**
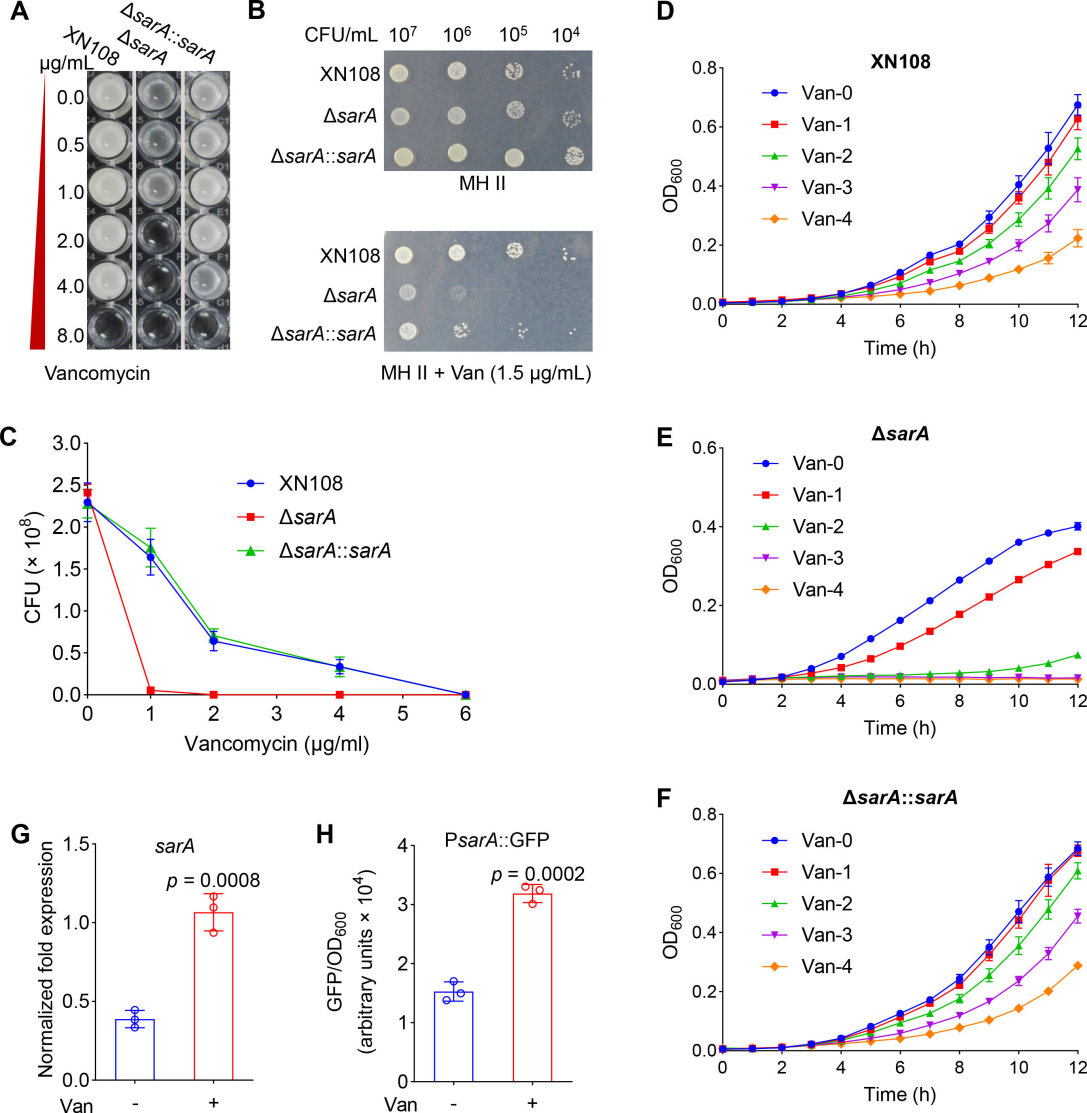
Disruption of *sarA* increases the susceptibility of *S. aureus* to vancomycin. (**A**) MICs of wild-type *S. aureus* XN108, Δ*sarA*, and chromosomal-complemented strain. All the strains were grown under vancomycin conditions in a 96-well plate with constant shaking at 200 rpm for 48 h at 37°C. (**B**) Detection of vancomycin sensitivity by using a plate-sensitivity assay. (**C**) Population analysis of the vancomycin susceptibility profiles of XN108, Δ*sarA*, and Δ*sarA::sarA*. MH II agar supplemented with vancomycin at concentrations of 0, 1, 2, 4, and 6 μg/mL was used to culture the strains, and the number of colonies was enumerated after incubation at 37°C for 48 h. The data are represented as the means ± SD. (**D–F**) growth curves of XN108, Δ*sarA*, and Δ*sarA::sarA*. MH II broth medium containing different concentrations of vancomycin (0, 1, 2, 3, and 4 μg/mL) was used to culture the target strains. The cultures were incubated in 96-well plates with an initial OD_600_ of 0.05 at 37°C under constant shaking at 200 rpm. The data are represented as the means ± SD. (**G**) The expression of *sarA* was induced by vancomycin. (**H**) Activity determination of *sarA* promoter. For panels **G and H**, the wild-type strain (**G**) or the fluorescent reporter strain (PALC-P*_sarA_*::GFP) (**H**) was incubated with (**+**) or without (−) vancomycin (Van, 80 μg/mL) at 37°C for 15 min. All experiments were performed in triplicate. The data are represented as the means ± SD. Statistical significance was determined by the two-tailed unpaired Student’s *t*-test.

Antibiotics are the main treatment options for *S. aureus* infection, and vancomycin has been used as the last-resort drug in such cases (7, 29). SarA, as a global regulator, has been reported to play a crucial role in regulating the virulence of *S. aureus* (8). However, a few reports have indicated that SarA regulates bacterial resistance to vancomycin. To explore the molecular mechanism of SarA in the regulation of the resistance of *S. aureus* to vancomycin, we constructed a *sarA* gene-knockout mutant in the VISA strain XN108 (30) and determined the sensitivity of the mutant to vancomycin. The results showed that, compared to the wild-type (XN108), the disruption of *sarA* increased the susceptibility of *S. aureus* to vancomycin (minimum inhibitory concentration [MIC] = 8 µg/mL for XN108 and MIC = 2 µg/mL for Δ*sarA*), and the MIC of the chromosome-complemented strain (Δ*sarA::sarA*) was consistent with that of the wild-type (Fig. 1A). Plate culture results also exhibited that the disruption of *sarA* decreased resistance to vancomycin in *S. aureus* (Fig. 1B). Growth curve assays and population analysis revealed that *sarA* deletion significantly inhibited the growth and survival of *S. aureus* in the presence of vancomycin (Fig. 1C through F). Moreover, the results of RT-qPCR and promoter activity detection revealed that the transcription of *sarA* was induced by vancomycin (Fig. 1G and H). Interestingly, knockout *sarA* in another VISA strain, Mu50, also showed increased sensitivity to vancomycin, which is consistent with the results in XN108 (Fig. S1A). However, knocking out *sarA* in the methicillin-susceptible *S. aureus* (MSSA) strain NCTC8325 and methicillin-resistant *S. aureus* (MRSA) strain USA300 LAC did not change the susceptibility of the strains to vancomycin (Fig. S1B and C and Table S5). Taken together, these results suggest the involvement of SarA in the regulation of the resistance of VISA to vancomycin and also that this regulatory process is strain-dependent.

### SarA is involved in the regulation of the cell wall thickness and autolysis of *S. aureus*

**Fig 2:**
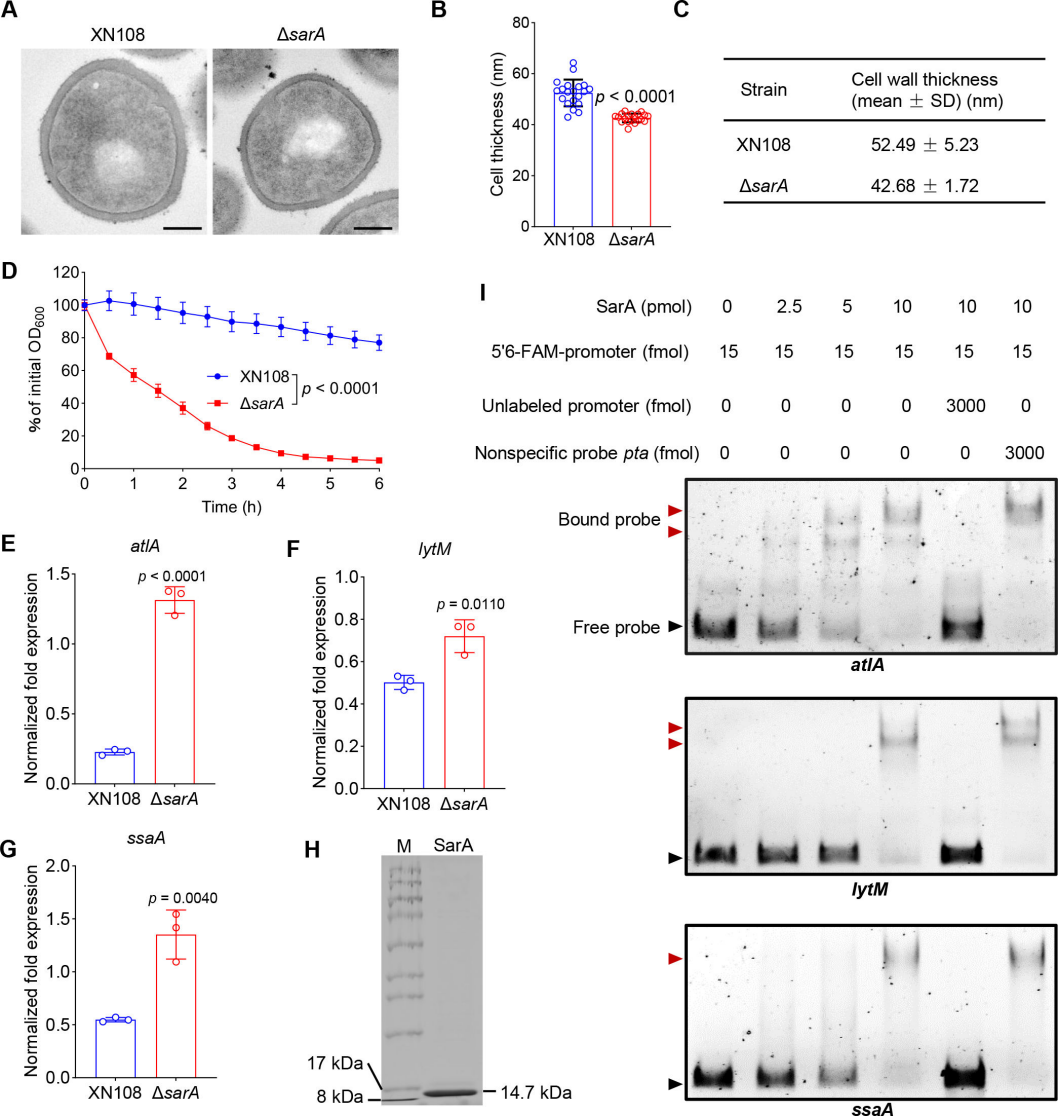
Deletion of *sarA* leads to the thinning of the cell wall and accelerated autolysis of *S. aureus*. (**A**) morphological features of XN108 and *sarA* gene-knockout mutant under TEM. Scale bar = 200 nm. (**B, C**) Cell wall thickness detection. The data are represented as the means ± SD for 50 cells of each strain. Each cell was measured thrice randomly by ImageJ. Statistical significance was determined by the two-tailed unpaired Student’s *t*-test. (**D**) Triton X-100-induced autolysis of XN108 and Δ*sarA*. The data are represented as the means ± SD. Statistical significance was determined using the two-sided two-way ANOVA. (**E–G**) Detection of autolysis-related gene expression in XN108 and Δ*sarA*. All experiments were performed in triplicate. The data are represented as the means ± SD. Statistical significance was determined by the two-tailed unpaired Student’s *t*-test. (**H, I**) Detection of the efficacy of SarA binding to the promoters of *atlA*, *lytM*, or *ssaA* by EMSA. The promoters were labeled with 6-FAM, and *pta* was used as a nonspecific competitor.

VISA strains typically exhibit thickened cell walls, which allow for increased trapping of vancomycin molecules by free D-Ala-D-Ala residues, thereby protecting cells from being killed by vancomycin (29). We therefore tested the cell wall thickness of XN108 and the *sarA* mutant via transmission electron microscopy (TEM), and the results showed that the cell wall thickness of the *sarA* mutant (42.14 ± 4.28 nm) was significantly reduced compared with that of XN108 (47.79 ± 7.01 nm) (Fig. 2A through C), indicating that SarA is involved in the regulation of the cell wall thickness. However, several genes related to cell wall synthesis (e.g., *ddl*, *dtlA*, *pbp2*, *scdA*, and *glyS*) were not significantly downregulated upon *sarA* deletion (Fig. S2A through E), suggesting that SarA may not increase cell wall thickness by regulating cell wall synthesis. We next explored whether SarA controls the cell wall thickness by regulating autolysis, and the results showed that the autolysis rate of the *sarA* mutant significantly increased compared with that of the wild-type (Fig. 2D). Correspondingly, the transcription levels of several autolysin-coding genes, such as *atlA*, *lytM*, and *ssaA*, were significantly elevated in Δ*sarA* compared to the wild-type (Fig. 2E through G). Moreover, the overexpression of *sarA* significantly inhibited the transcription of these genes (Fig. S3A through D). However, the expression levels of other autolysis-related genes, such as *isaA* and *sceD*, were significantly reduced (Fig. S2F and G). The results of the electrophoretic mobility shift assay (EMSA) showed that SarA could directly regulate the expression of *atlA*, *lytM*, and *ssaA* by binding to their promoters (Fig. 2H and I). Based on these data, we concluded that SarA could regulate the cell wall thickness of *S. aureus* by regulating autolysis.

### The resistance of *S. aureus* to vancomycin regulated by SarA is partly dependent on ABC-like

**Fig 3:**
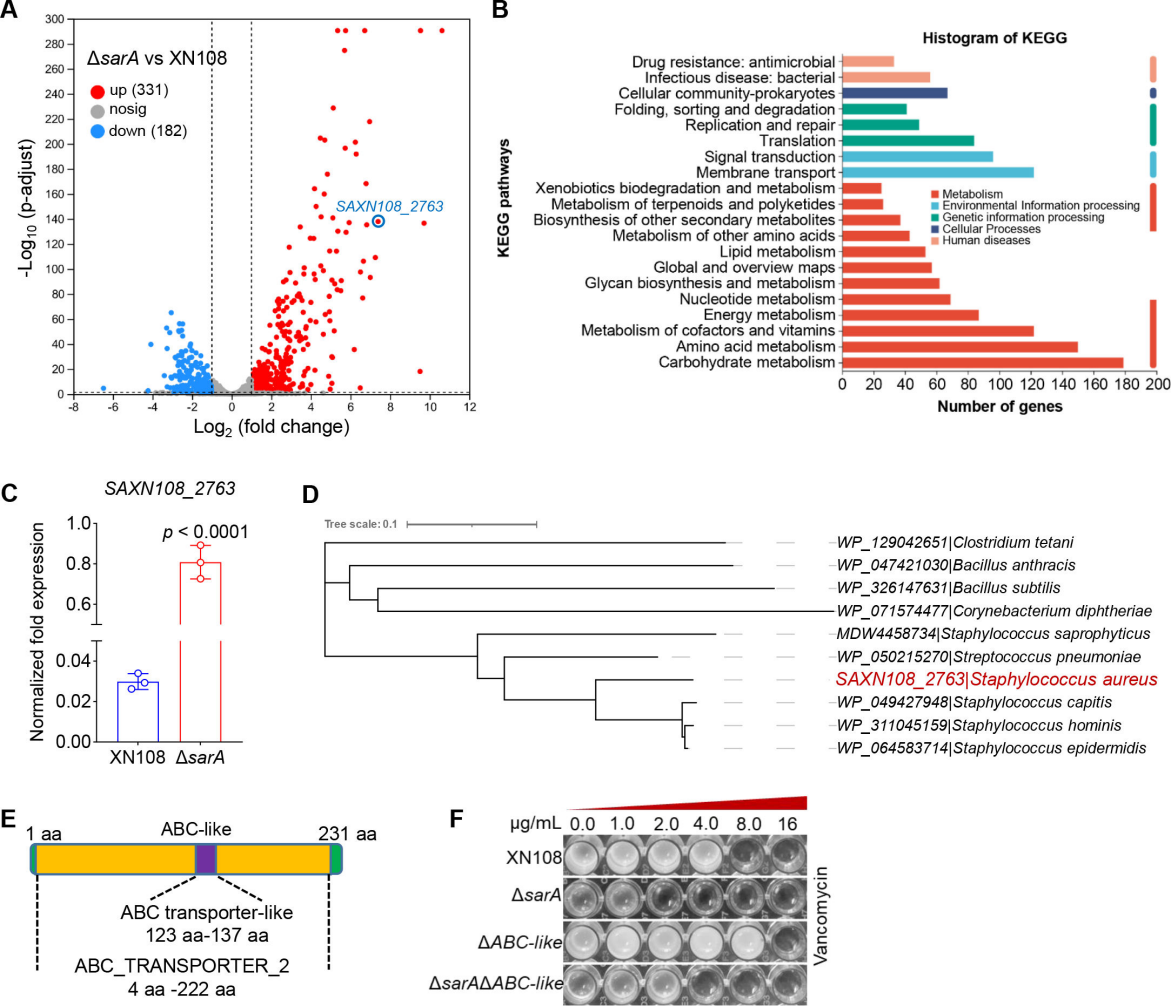
SarA modulates the resistance of *S. aureus* to vancomycin by negatively regulating *ABC-like* expression. (**A**) "Volcano map" of differentially expressed genes in XN108 and Δ*sarA*. (**B**) KEGG pathway map of the significantly altered genes between XN108 and Δ*sarA*. (**C**) Transcriptional level of *SAXN108* in XN108 and Δ*sarA*. The experiment was performed in triplicate. The data are represented as the means ± SD. Statistical significance was determined by the two-tailed unpaired Student’s *t*-test. (**D**) Cluster analysis for SAXN108. The accession numbers of WP_129042651, WP_047421030, WP_326147631, WP_071574477, MDW4458734, WP_050215270, WP_049427948, WP_311045159, and WP_064583714 are all sourced from the NCBI database, and all the proteins belong to the ABC protein family. (**E**) Structural prediction of SAXN108_2763. (**F**) MICs of wild-type *S. aureus* XN108, Δ*sarA,* Δ*ABC-like*, and Δ*sarA*Δ*ABC-like*. All the strains were grown under vancomycin conditions in a 96-well plate under constant shaking at 200 rpm for 72 h at 37°C.

RNA-seq was performed to investigate whether SarA could regulate the resistance of *S. aureus* to vancomycin by regulating the expression of genes other than those associated with autolysis and cell wall synthesis. The results indicated that the gene expression profiles of *S. aureus* XN108 and Δ*sarA* strains were considerably different, with 331 upregulated and 182 downregulated genes, and these significantly altered genes were involved in antibiotic stress resistance, bacterial infection, and signal transduction (Fig. 3A and B; Table S3). Among the significantly altered genes, the upregulated gene SAXN108_2763 was noted in the *sarA* mutant relative to that in XN108, as confirmed by RT-qPCR (Fig. 3A and C; Table S3). Cluster analysis indicated that the protein belongs to the ABC family (Fig. 3D). Further analysis revealed that the protein contained conserved regions of the ABC transporter, except for the TMDs, hence named ABC-like (Fig. 3E). Moreover, the ABC transport systems in bacteria are reportedly involved in drug resistance (26, 31). We then investigated whether ABC-like was involved in regulating the resistance of *S. aureus* to vancomycin. We constructed a single-knockout mutant of *ABC-like* and a double-knockout mutant of *ABC-like* and *sarA*. The MIC test results revealed that, compared with XN108 (8 μg/mL), the *ABC-like* mutant showed increased resistance to vancomycin (MIC = 16 µg/mL) (Fig. 3F). Cumulatively, these results suggest that SarA can regulate the resistance of *S. aureus* to vancomycin by negatively regulating *ABC-like* expression.

### Vancomycin promotes the direct regulation of SarA on the transcription of *ABC-like*

**Fig 4:**
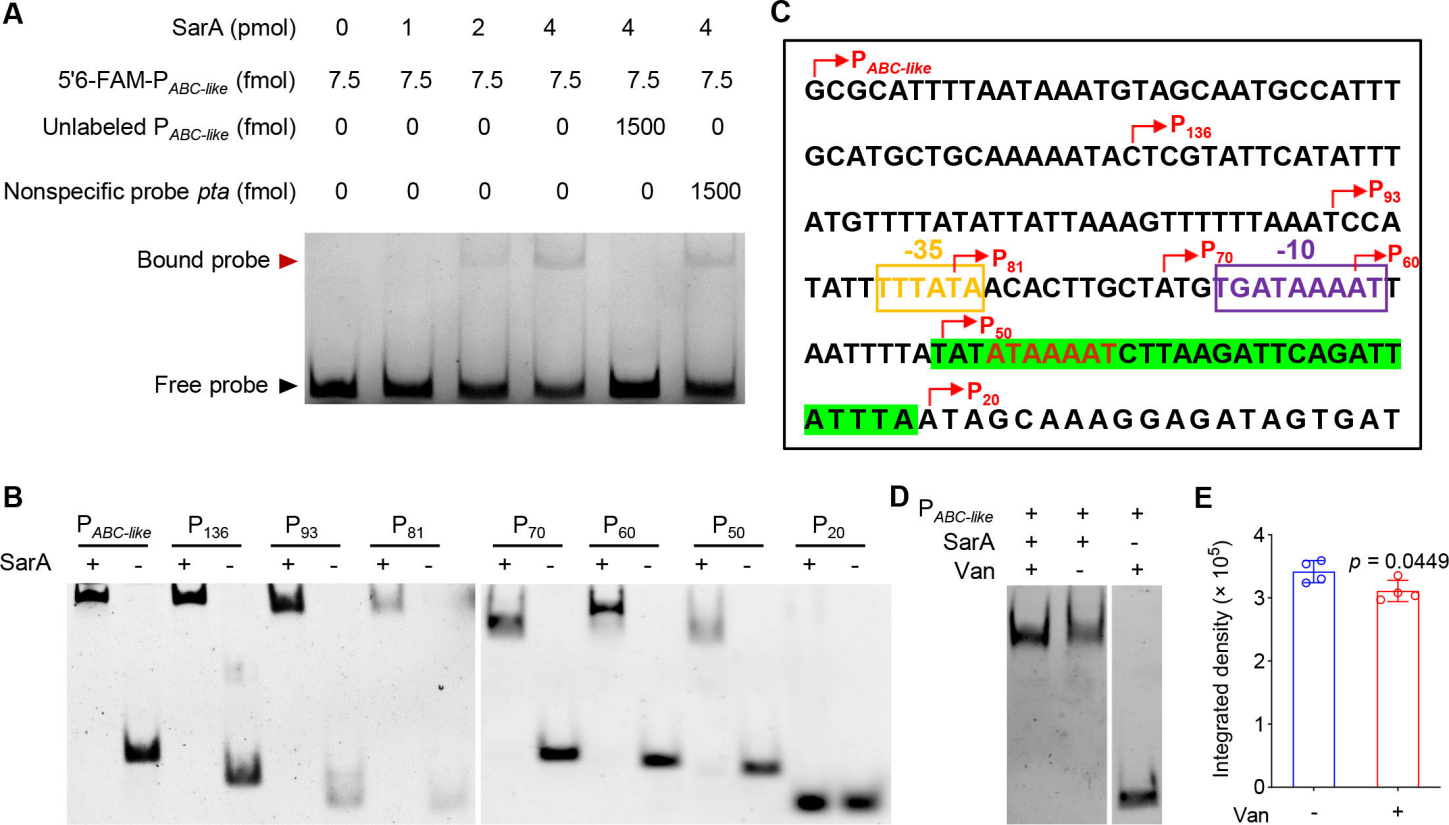
SarA directly regulates the expression of *ABC-like*. (**A**) EMSA of the binding of SarA to the promoter of the *ABC-like* gene. The 6-FAM fluorescent probe was used to label the promoter. The unlabeled probe was then added as a specific competitor, and the unlabeled fragment of the *pta* was added as a nonspecific competitor. (**B**) EMSA analysis of the core region of the *ABC-like* promoter binding to SarA. SarA: 80 pmol, *ABC-like*: 7.5 fmol. (**C**) The promoter sequence of the *ABC-like* gene. The truncated positions of the promoter are indicated by red arrows. The −10 and −35 regions (analyzed by BPROM) of the *ABC-like* promoter are highlighted with a yellow box and a bluish-violet box, respectively, while the core region binding to SarA is marked with a green background. The SarA binding site, as defined by Sterba et al. (32) (5′ATTTTAT3′), is shown with red letters (reverse complementary sequences). (**D, E**) Vancomycin promotes the binding of SarA to the *ABC-like* promoter. The binding of SarA to ABC-like was assessed by EMSA under conditions with (+) or without (−) vancomycin. The binding intensity was analyzed using ImageJ software. The data are presented as the means ± SD. Statistical significance was determined using the two-tailed unpaired Student’s *t*-test.

To reveal the mechanism underlying the altered expression of *ABC-like*, EMSA was performed to verify whether the transcription of *ABC-like* is under the direct control of SarA. The results showed that SarA could retard the mobility of the putative promoter region (183 bp) of the *ABC-like* gene in a dose-dependent manner (Fig. 4A), implying that SarA, which serves as a repressor for *ABC-like*, modulates the transcription of *ABC-like* by directly binding to its promoter. We next attempted to determine the binding sites of SarA on the *ABC-like* promoter by creating a series of truncated versions of the *ABC-like* promoter. The results showed that SarA could bind to P136, P93, P81, P70, P60, and P50, but not to P20, indicating that the P50–20 region is critical for SarA binding to the *ABC-like* promoter (Fig. 4B). Moreover, the region of P50–20 contains the DNA motif 5′ATTTTAT3′ (reverse complementary sequence) that has been reported to bind SarA (32) (Fig. 4C). We also found that vancomycin could promote the binding of SarA to the ABC-like promoter (Fig. 4D and E). Taken together, these results suggest that vancomycin enhances the direct regulation of ABC-like transcription by SarA, implying that SarA may increase *S. aureus* resistance to vancomycin by directly repressing the expression of the *ABC-like* gene.

### ABC-like can bind to vancomycin and regulate the surface charge of *S. aureus*

**Fig 5:**
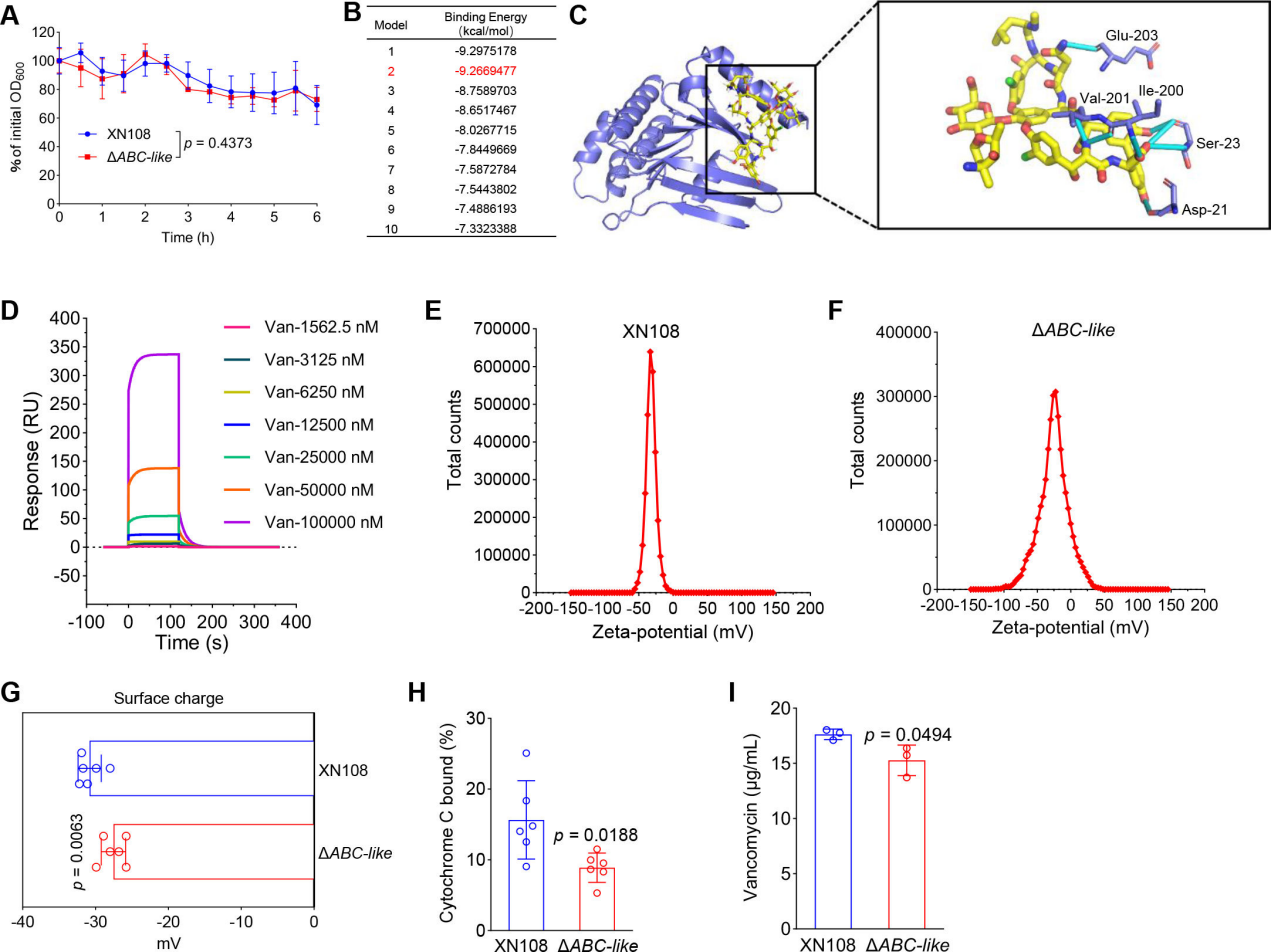
Disruption of *ABC-like* significantly reduces vancomycin binding to *S. aureus*. (**A**) Autolytic activity assay of XN108 and Δ*ABC-like*. The data are presented as the mean ± SD. Statistical significance was determined by two-sided two-way ANOVA. (**B, C**) Molecular docking prediction of vancomycin binding to ABC-like. MOE software was used to analyze the binding mode of vancomycin with ABC-like, and the docking-binding energy estimation of −9.267 kcal/mol was selected for subsequent analyses (**B**). Vancomycin is displayed as a yellow ball-and-stick model, while ABC-like is depicted as a purple cartoon model. The dark blue color represents nitrogen atoms, red represents oxygen atoms, green represents chlorine atoms, and light blue indicates the formed hydrogen bonds (**C**). The binding diagram was created using PyMOL. (**D**) The binding of vancomycin and ABC-like protein was detected by SPR. (**E–G**) The detection of the net surface charge on *S. aureus*. The net surface charge was determined using a zeta potential analyzer. (**H**) The binding of cytochrome C to whole *S. aureus* cells. Cytochrome C (0.5 mg/mL) and *S. aureus* cells were incubated at room temperature for 30 min, followed by centrifugation and measurement of the OD_530_ value of the supernatant. (**I**) Detection of the vancomycin binding capacity of XN108 and the *ABC-like* gene-knockout mutant. The strains were treated with vancomycin (40 μg/mL) for 1 h with constant shaking at 220 rpm and 37°C. Vancomycin in the supernatant of lysed *S. aureus* cells was detected by HPLC. For panels **G–I**, the data are presented as the means ± SD. Statistical significance was determined using the two-tailed unpaired Student’s *t*-test.

Our results have demonstrated that SarA can regulate the resistance of *S. aureus* to vancomycin by modulating autolysis. Given that SarA can directly regulate the expression of the ABC-like gene by binding to its promoter, we hypothesized that SarA modulates the resistance of *S. aureus* to vancomycin through direct transcriptional regulation of *ABC-like*. To this end, we examined the autolytic activity of the *ABC-like* gene-knockout mutant, and the results showed that there was no significant difference in autolytic activity between XN108 and Δ*ABC-like* (Fig. 5A), indicating that SarA does not regulate vancomycin sensitivity in *S. aureus* by modulating *ABC-like* expression to affect autolytic activity. Previous studies have shown that ABC transporters can bind and transport substrates, thereby playing a role in regulating bacterial drug resistance (33). We employed MOE to perform molecular docking to predict the binding between vancomycin and ABC-like. To ensure the reliability of the docking results, we selected the conformation with the lowest binding free energy and the second-best scoring conformation, in which vancomycin displayed a significant interaction with ABC-like (predicted docking binding energy = −9.267 kcal/mol) for further analysis (Fig. 5B). The analysis showed that vancomycin forms one hydrogen bond with the backbone oxygen atom of Gln203 in ABC-like Chain A, one hydrogen bond with the side-chain oxygen atom of Asp21, three hydrogen bonds with the backbone nitrogen and oxygen atoms of Ser23, two hydrogen bonds with the backbone nitrogen and oxygen atoms of Ile200, and one hydrogen bond with the backbone oxygen atom of Val201 (Fig. 5C). We next validated the results using surface plasmon resonance (SPR), which demonstrated that ABC-like can bind to vancomycin (Fig. 5D and Table S4), suggesting that ABC-like may enhance *S. aureus* sensitivity to vancomycin by binding the drug.

Furthermore, as vancomycin is a positively charged glycopeptide targeting the cell wall of gram-positive bacteria and the bacterial surface is negatively charged, we sought to demonstrate that ABC-like can alter the surface charge of *S. aureus* to control the binding of vancomycin to the bacterial cell wall. Zeta potential analysis revealed that the surface negative charge of the *ABC-like* gene-knockout mutant was significantly reduced compared to that of XN108 (Fig. 5E through G). We also assessed the binding capacity of *S. aureus* to cytochrome C (a highly charged molecule, pI = 10) to characterize the net surface charge of *S. aureus*. The results showed that disrupting *ABC-like* significantly reduced the binding capacity of *S. aureus* to cytochrome C (Fig. 5H), again indicating a significant reduction in the surface negative charge of the *ABC-like* gene-knockout mutant. These may lead to a reduced efficiency of vancomycin binding to the cell wall, thereby increasing the resistance of the *ABC-like* gene-knockout mutant to vancomycin. Concurrently, we measured the amount of vancomycin bound to *S. aureus*, and the results showed that the amount of vancomycin bound to the Δ*ABC-like* mutant was significantly lower than that in XN108 (Fig. 5I). In summary, the above results suggest that ABC-like regulates *S. aureus* resistance to vancomycin by binding vancomycin and altering the net surface charge of the bacterial cell.

### Vancomycin effectively inhibits the colonization of *sarA* gene-knockout mutant in mice

**Fig 6:**
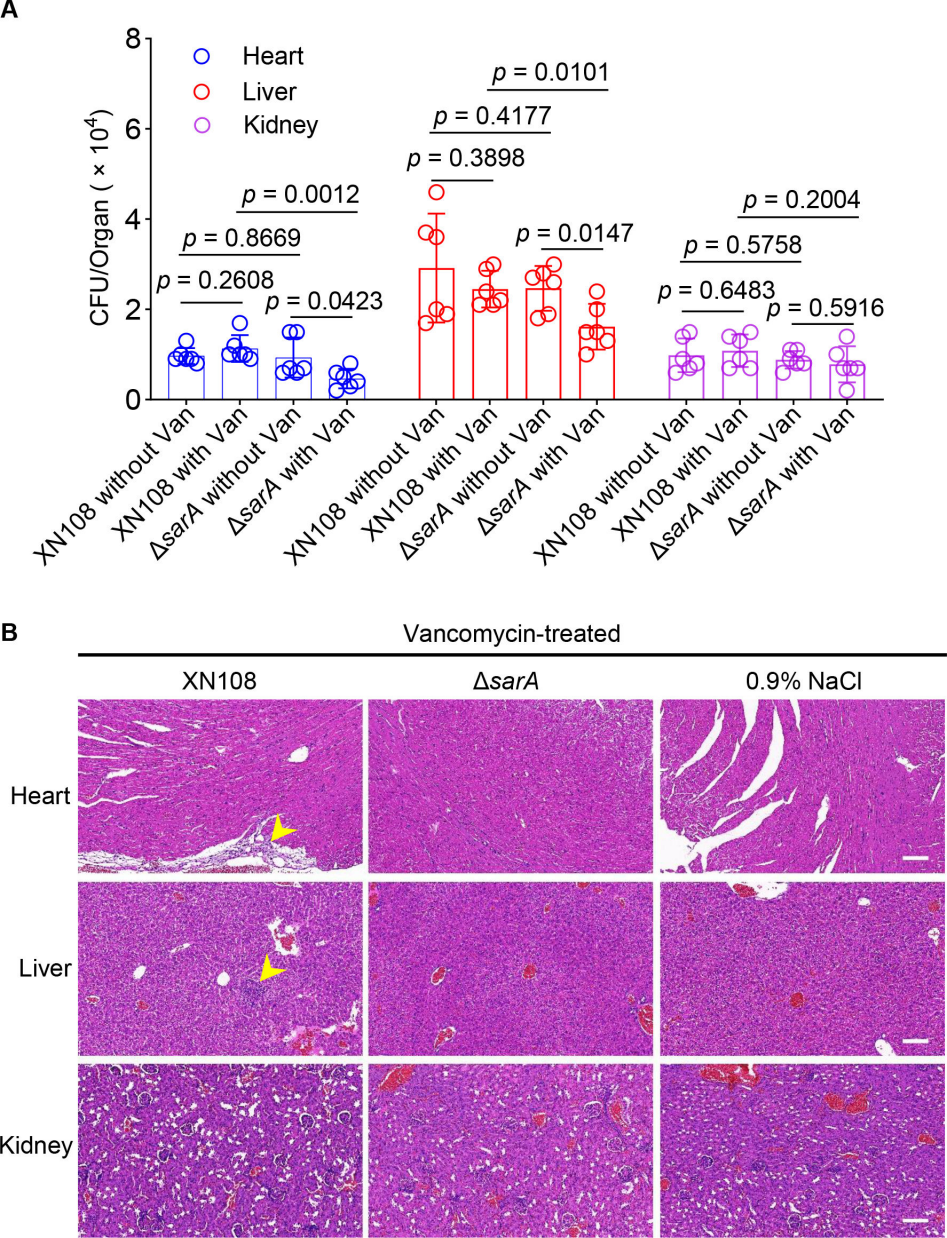
Disruption of *sarA* significantly reduces the survival rate of *S. aureus* in mouse organs. (**A**) Detection of the colonization levels of *S. aureus* in mouse organs. The mice were inoculated with 200 μL of 0.9% NaCl containing 1 × 10^8^ CFU of each target strain *via* intraperitoneal injection. Vancomycin was administered intraperitoneally at a dose of 32 mg/kg daily (24 h intervals) for 4 consecutive days. The data are presented as the means ± SD (*n* = 6). Statistical significance was determined using the two-tailed unpaired Student’s *t*-test. (**B**) H&E staining of the mouse organs. After the infection of mice with *S. aureus*, continuous treatment with vancomycin for 4 days was administered. The organs of the mice were then collected for H&E staining analysis. The yellow arrowheads indicate mild inflammatory cell infiltration.

We demonstrated that disrupting *sarA* significantly increased the susceptibility of *S. aureus* to vancomycin (MIC = 8 µg/mL for XN108 and MIC = 2 µg/mL for Δ*sarA*). To further validate the inhibitory effect of vancomycin on the *sarA* mutant, we introduced a mouse infection model. After infecting the mice with *S. aureus*, the mice were treated with vancomycin or 0.9% NaCl, and the bacterial colonization levels in the internal organs were quantified. In addition, the histological structure of the organs was observed. The results revealed that, without vancomycin treatment, there was no significant difference in the colonization numbers of the wild-type *S. aureus* XN108 and the *sarA* mutant in the heart, liver, or kidney of the mice (Fig. 6A). In the heart and liver, the colonization numbers of XN108 did not significantly change before and after vancomycin treatment. However, the colonization numbers of the *sarA* mutant significantly decreased under vancomycin treatment, albeit this phenomenon was not observed in the kidney (Fig. 6A). Hematoxylin-eosin (H&E) staining results showed that, after vancomycin treatment, mild leukocyte infiltration remained in the heart and liver of the mice infected with XN108, whereas no such phenomenon was observed in the mice infected with the *sarA* mutant (Fig. 6B). Moreover, no significant difference was noted in the kidney structure of the mice infected with either XN108 or the *sarA* mutant after vancomycin treatment (Fig. 6B). Altogether, these findings indicate that vancomycin can effectively inhibit the growth of Δ*sarA* in mice, thereby further illustrating the regulatory role of SarA in vancomycin resistance in *S. aureus*.

## DISCUSSION

**Fig 7:**
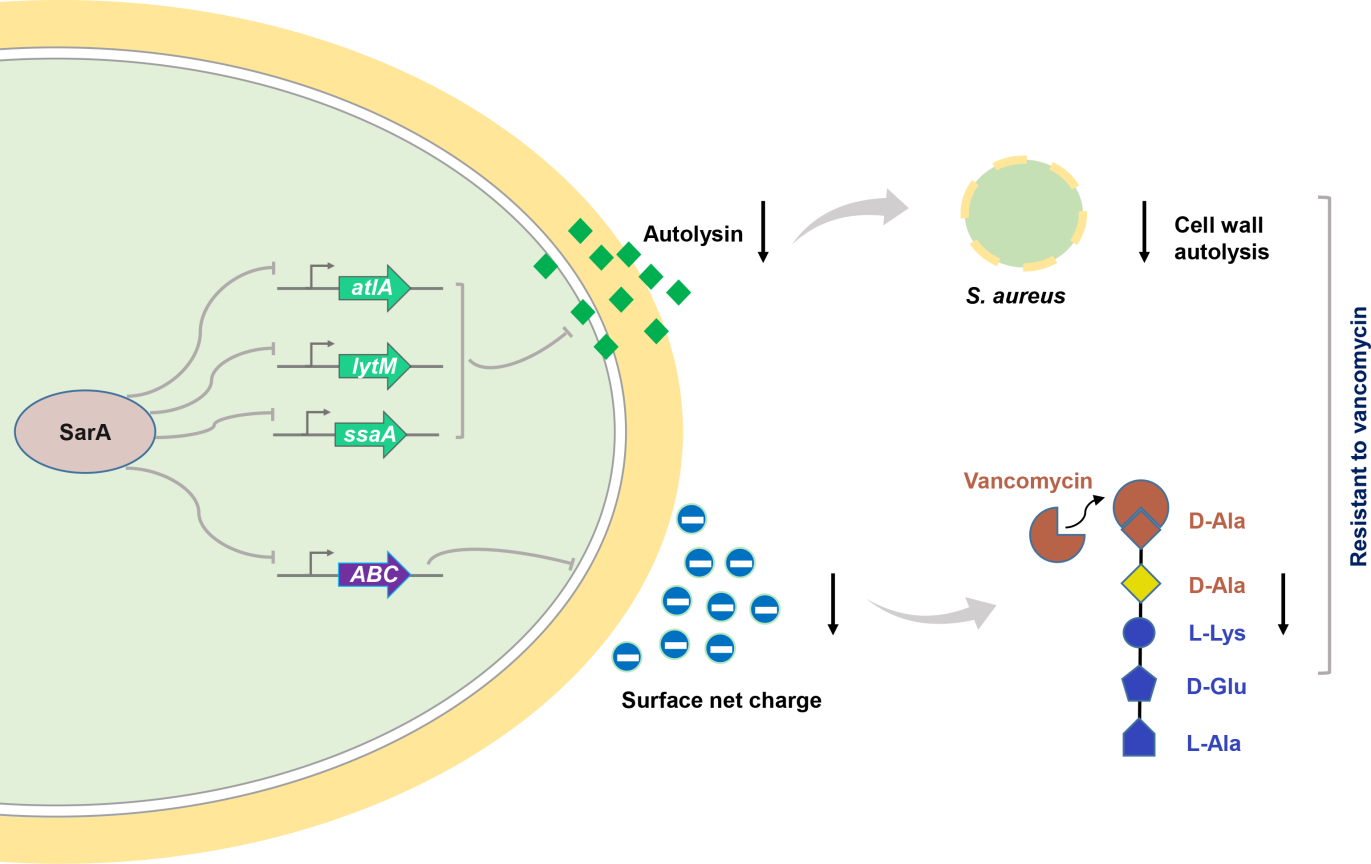
Schematic diagram of SarA regulating vancomycin resistance in *S. aureus.* SarA reduces cell wall autolysis by directly inhibiting the expression of autolysis-related genes such as *atlA*, *lytM*, and *ssaA*. Concurrently, it decreases the transcription of *ABC-like*, which lowers the net surface charge of *S. aureus*, thereby reducing the binding efficiency of vancomycin to the cell wall. Ultimately, this results in reduced sensitivity of *S. aureus* to vancomycin.

*S. aureus*, a gram-positive opportunistic and notorious pathogen, is responsible for a wide range of infections, both community- and hospital-acquired, such as bacteremia, pneumonia, sepsis, and osteomyelitis (34, 35). The emergence of multidrug-resistant and highly virulent strains of *S. aureus* has posed significant challenges in treating these infections. Investigating the drug resistance mechanisms of *S. aureus* is essential for advancing effective therapeutic approaches to combat infections caused by this pathogen. Here, we have demonstrated that SarA mediates the vancomycin resistance of *S. aureus* by directly negatively regulating the expression of autolysis-related genes. Furthermore, SarA can negatively regulate the expression of *ABC-like*, altering the net surface charge of *S. aureus*, thereby affecting the efficiency of vancomycin binding to the bacterium and ultimately reducing the sensitivity of *S. aureus* to vancomycin (Fig. 7). This study provides valuable insights for the clinical treatment of VISA and other antibiotic-resistant pathogens and offers important perspectives for the clinical management of VISA and other antibiotic-resistant infections.

The SarA protein family forms a crucial regulatory network in *S. aureus*, participating in the responses to diverse environmental conditions (36–38). As a global regulatory factor, SarA is involved in modulating the virulence and biofilm formation of *S. aureus* (8, 9). In addition, although it is known that SarA can regulate the autolysis activity of *S. aureus* (39), the underlying specific mechanism remains unexplored. Our findings indicate that disrupting *sarA* can significantly increase the expression levels of the autolysis-related genes *atlA*, *lytM*, and *ssaA* (Fig. 2E through G). However, the transcriptional levels of other autolysis-related genes, such as *isaA* and *sceD,* did not significantly increase upon *sarA* gene knockout (Fig. S2F and G). Moreover, we assessed the expression of genes related to cell-wall synthesis and found that the expression of these genes (e.g., *ddl*, *dtlA*, *pbp2*, *scdA*, and *glyS*) was not significantly reduced in the *sarA* gene-knockout background (Fig. S2A through E). Combined with the observation that the cell wall of the Δ*sarA* mutant was significantly thinner (Fig. 2A through C), these results suggest that SarA regulates *S. aureus* autolysis activity, rather than cell-wall synthesis, leading to a thinner cell wall, ultimately modulating *S. aureus* sensitivity to cell wall-targeting antibiotics such as vancomycin.

ABC transporters are one of the largest protein superfamilies and are found in all living organisms (22). They are crucial for numerous cellular functions, exhibit a conserved structural design, and operate through a common mechanism that couples ATP hydrolysis to the transport of substrates (40). In this study, we found that SarA can directly bind to the promoter region of *ABC-like*, negatively regulating its expression, with the core binding site identified as 5′ATTTTAT3′ (Fig. 4A through C). We also discovered that disrupting ABC-like significantly reduces the surface negative charge of *S. aureus* (Fig. 5E through G). Since vancomycin is a positively charged glycopeptide antibiotic, the reduction in the surface negative charge of *S. aureus* may decrease its affinity for vancomycin, which could explain the observed significant reduction in the susceptibility of *S. aureus* to vancomycin (Fig. 3F). Our findings provide new insights into understanding how SarA regulates antibiotic resistance in *S. aureus*. However, the specific mechanism by which SarA regulates the net surface charge of *S. aureus* through ABC-like remains to be further explored.

In this study, cluster analysis revealed that ABC-like belongs to the ABC family (Fig. 3D). However, structural predictions indicated that ABC-like lacks TMDs (Fig. 3E). We next attempted to determine the localization of ABC-like but were unsuccessful. It has been reported that, unlike traditional ABC transporters, ABC-F proteins are not membrane-bound; nevertheless, they contribute to the regulation of antibiotic resistance (28). Therefore, we hypothesize that ABC-like may belong to the ABC-F proteins or function as an ABC-transporter component. Furthermore, although our results demonstrated that disruption of *ABC-lik*e significantly reduced the surface negative charge of *S. aureu*s (Fig. 5E through G), we did not prove that ABC-like directly alters the surface charge of *S. aureus*. Given the absence of TMDs in ABC-like, it is unlikely to modify the surface charge through direct transport of charged precursor molecules. We speculate that ABC-like may indirectly influence the surface charge of *S. aureus* via alternative regulatory pathways, such as modulating the expression or activity of enzymes involved in cell wall synthesis or modification. However, the specific mechanisms remain to be further elucidated.

To verify whether SarA regulates the vancomycin resistance in *S. aureus* in a strain-dependent manner, we constructed *sarA* gene*-*knockout mutants in the VISA strain Mu50, the MRSA strain USA300 LAC, and the MSSA strain NCTC8325, and then assessed their vancomycin resistance. The results revealed that the knockout of *sarA* in Mu50 significantly increased the susceptibility of *S. aureus* to vancomycin, but not in USA300 LAC and NCTC8325 (Fig. S1A through C). Owing to the limited sample size, these findings require further investigation. Nonetheless, our data suggest, to some extent, that SarA-mediated regulation of antibiotic resistance in *S. aureus* is strain-dependent.

The *in vivo* experimental results in mice showed that, under vancomycin treatment, the knockout of *sarA* significantly reduced the colonization ability of *S. aureus* in the heart and liver, and compared to Δ*sarA*, mice infected with XN108 displayed mild inflammatory responses in the heart and liver (Fig. 6A and B). However, this phenomenon did not occur in the kidneys of the mice. The exact reason for this remains to be further investigated. In any case, this study demonstrates that vancomycin has a stronger inhibitory effect on the *sarA* mutant than on the wild-type, both *in vivo* and *in vitro*, confirming the regulatory role of *sarA* in *S. aureus* resistance.

In summary, we demonstrated that SarA, on one hand, can suppress the expression of autolysis-related genes to regulate the susceptibility of *S. aureus* to vancomycin, and on the other hand, can negatively regulate the expression of *ABC-like* to alter the surface charge of *S. aureus*, thereby influencing its resistance to vancomycin. Our findings thus imply the potential of SarA to serve as a therapeutic target for treating *S. aureus* infections.

## MATERIALS AND METHODS

### Strains, plasmids, and culture conditions

*S. aureus* strains were grown in tryptic soy broth (TSB) (211825, BD-Difco) or tryptic soy agar (TSA) medium at 37°C. *Escherichia coli* DH5α (Yeasen, 11803ES80) and BL21 (Weidi, EC1002) were used for routine DNA manipulations and protein expression, respectively, and their derived strains were grown in lysogeny broth (LB) or lysogeny broth agar (LA) medium at 37°C. As required, 60 μg/mL ampicillin sodium salt and 15 μg/mL chloramphenicol were added to TSB/TSA and LB/LA media, respectively. Strains and plasmids used in this study were listed in Table S1.

### DNA manipulation

The primers used in this study are listed in Table S2. The pBTs vector was used for *sarA* gene-knockout mutant construction as described previously (13, 41). All target PCR products were acquired using PrimeSTAR MAX Premix (TaKaRa, R045). For the construction of the *sarA* gene-deletion vector, 5′-end 808-bp and 3′-end 802-bp fragments of the *sarA* gene flanking sequences were cloned from *S. aureus* XN108 genomic DNA via PCR using primer pairs *sarA*-LB-F/*sarA*-LB-R and *sarA*-RB-F/*sarA*-RB-R, respectively. The 5′- and 3′-end PCR products were integrated by overlapping PCR and then inserted into pBTs using T4 DNA ligase (Thermo Scientific, EL0011) to generate the vector pBTs-LB-*sarA*-RB. The allelic replacement mutant was selected via previously described protocols (13, 42) and was further verified by PCR and sequencing. A similar strategy was used to construct the *sarA* complementary strain in Δ*sarA*, the *ABC-like* mutant in XN108, as well as the *sarA* mutant in Mu50.

For *sarA* overexpression, the target fragment was cloned from XN108 genomic DNA with the primers *sarA*-OE-F and *sarA*-OE-R, and the PCR product was then inserted into pLI50 to form pLI50-*sarA*-OE.

For GFP reporter vector construction, the *sarA* promoter (305 bp) was cloned from XN108 genomic DNA with the primer pair P*_sarA_*_-Ac_-F/P*_sarA_*_-Ac_-R, and the fragment was then inserted into the modified PALC-GFP (the coding sequences of GFP were amplified from plasmid eGFP-C1 [27]) vector to form PALC-P_*sarA*_::GFP.

All the vectors were transformed into *S. aureus* RN4220 for modification and were subsequently transformed into the target strains for subsequent analysis.

### RNA-seq

For RNA-seq, overnight cultures of *S. aureus* wild-type XN108 and Δ*sarA* were diluted in TSB (OD_600_ ≈ 0.05) and allowed to grow to mid-logarithmic phase. Three clones from each strain were selected for subsequent analysis. The cells were collected by centrifugation (12,000 × *g*, 2 min) and sent to Major Biotechnology Co., Ltd. (Shanghai, China) for RNA-seq analysis. The transcriptome library construction and subsequent sequencing analysis were carried out according to our previous studies (13). The RNA-seq transcriptome library was constructed with the Illumina TruSeq RNA Sample Preparation Kit (RS-122-2001), starting with 2 μg of total RNA. Ribosomal RNA was removed using the Ribo-Zero Magnetic Kit (MRZB12424, Epicenter) as an alternative to poly(A) selection. Subsequent sequencing data generated on the Illumina platform were subjected to bioinformatics analysis on the Majorbio Cloud Platform (www.majorbio.com). The analytical steps included: mapping high-quality reads from each sample to the reference genome with Bowtie2; quantifying gene and isoform abundance using RSEM; and identifying differentially expressed genes (DEGs) utilizing edgeR, DESeq2, or DESeq. Furthermore, significantly enriched Gene Ontology terms among the DEGs were identified using Goatools via Fisher’s exact test, with terms possessing an adjusted *P*-value ≤0.05 after multiple testing correction deemed statistically significant. The raw data of RNA-seq have been uploaded to NCBI (https://www.ncbi.nlm.nih.gov/sra/PRJNA1104410; BioProject accession number: PRJNA1104410).

### Gene expression analysis

The overnight cultures of the target strains were diluted in TSB at an initial OD_600_ of 0.05 and cultivated to mid-logarithmic phase. The cells were then collected via centrifugation (12,000 × *g*, 2 min). The total RNA of *S. aureus* was extracted using RNAiso Plus (TaKaRa, AKF0727A), as described previously (43). About 1 μg of total RNA was used for cDNA synthesis via RT-PCR using the PrimeScript RT Reagent Kit (TaKaRa, RR047A). Quantitative RT-PCR (RT-qPCR) was performed with 2 × RealStar Fast SYBR (GenStar, A301-10). The total volume of the PCR system was 10 µL, including 5 µL 2 × RealStar Green Fast Mixture, 0.5 µL upstream and downstream primers (10 µmol), and 1 µL cDNA. *pta* (gene ID: 3913780) was used as the internal reference. The PCR cycle conditions were as follows: 95°C for 2 min, then 95°C for 15 s and 60°C for 30 s for 40 cycles. The melting curve analysis was carried out to guarantee the quality. The relative expression of the target genes was normalized to that of the internal reference gene (normalized fold expression), and CFX Manager 3.1 software (Bio-Rad) was used for processing. All RT-qPCR primers are listed in Table S2.

### Antibiotic sensitivity assays

The MICs of vancomycin for the target *S. aureus* were performed in Mueller-Hinton II (MH II; Difco, 212322) medium by using broth microdilution technique according to NCCLS guidelines. The target strains were recovered on the TSA plate until colonies appeared, and three to four colonies of each strain were picked into 300 μL MH II medium and mixed thoroughly. Then the *S. aureus* cells were mixed with different concentrations of antibiotics and the final concentration of *S. aureus* cells was 5 × 10^5^ CFU/mL. The 96-well plates were cultured at 37°C with shaking at 200 rpm for 48–72 h.

For the plate-sensitivity assay, resuscitated *S. aureus* cells were collected from TSA plates and diluted to different concentrations (1 × 10^7^ CFU/mL, 1 × 10^6^ CFU/mL, 1 × 10^5^ CFU/mL, 1 × 10^4^ CFU/mL) with ddH_2_O, and 5 μL of the *S. aureus* cells were inoculated onto the agar plates of MH II medium, and the plates were cultured at 37°C for about 30 h.

### Growth curves

The method was conducted as previously described (44) with slight modification. Briefly, the strains of XN108, Δ*sarA*, and Δ*sarA::sarA* were cultured in TSB with shaking at 220 rpm and 37°C overnight and then the cultures were diluted with 150 μL fresh MH II broth medium containing different concentrations of vancomycin (0, 1, 2, 3, and 4 μg/mL) to achieve an initial OD_600_ of 0.05 in 96-well plates (200 rpm, 37°C). The OD_600_ was measured hourly using an ELx800 microplate reader (Bio-Tek).

### Fluorescence-dependent activity assay for *sarA* promoter

Fluorescence (GFP) reporting system was introduced to determine *sarA* promoter activity. Overnight cultures of XN108 were diluted in 20 mL TSB (OD_600_ ≈ 0.05) and allowed to grow to mid-logarithmic phase. Then the cultures were evenly divided into two triangulated bottles, one of which was added with vancomycin (80 μg/mL, 10 × MIC of XN108), and induced for 15 min. The samples were collected and washed twice with PBS and diluted in PBS at an initial OD_600_ of 0.5. GFP fluorescence intensity (excitation = 488 ± 9 nm, emission = 518 ± 20 nm) was determined using a CLARIOstar plate reader (BGM Labtech). *sarA* promoter activity was calculated according to the following formula: promoter activity = GFP fluorescence intensity/OD_600_.

### TEM

For morphological changes detection, the target strains were cultured in TSB at 37°C with shaking at 220 rpm and collected by centrifugation (12,000 × *g*, 2 min). Samples were prepared as previously described (29) and sent to the Cryo-EM Center at the University of Science and Technology of China for subsequent analysis. Specimens were examined with a TEM operated at an accelerating voltage of 200 kV.

### Triton X-100-induced autolysis assay

Overnight-grown cells of the target strains were diluted to an initial OD_600_ of 0.05 in TSB and grown to an OD_600_ of 0.7–0.8 at 37°C. The cells were collected at 8,000 rpm for 5 min, washed with Tris-HCl (50 mM, pH 7.5) thrice, and then suspended in Tris-HCl (50 mM, pH 7.5) containing 0.1% (vol/vol) Triton X-100. The cell suspensions of the target strains were cultured in 96-well plates at 37°C with shaking at 200 rpm. The autolysis was detected by measuring the progressive decrease in absorbance (OD_600_) at 30 min intervals by using a microplate reader (ELx800, Bio-Tek). The detection was stopped when the OD_600_ value was reduced to half of the initial value, and the autolysis ratio can be calculated by the following formula: current OD_600_ value/initial OD_600_ value.

### Protein expression and purification

The pET-28a (+) vector was used for protein expression. Overnight culture of *E. coli* BL21 (DE3) containing the target plasmid was diluted in LB with 50 μg/mL kanamycin at an initial OD_600_ of 0.05 and then grown to OD_600_ ≈ 0.6–0.8 and induced with 0.5 mM isopropyl-β-D-1-thiogalactopyranoside at 16°C for about 16 h. Protein purification was performed according to the His-tag Protein Purification Kit (Beyotime, P2226) instructions.

### EMSA

The 6-FAM-labeled putative promoters of the target genes (*ABC-like*, *altA*, *lytM*, and *ssaA*) were amplified from the genomic DNA of XN108. SarA protein and the labeled probes were incubated in binding buffer (20 μL, 20 mM HEPES, pH 7.6, 1 mM EDTA, 10 mM (NH_4_)_2_SO_4_, 30 mM KCl, 1 mM dithiothreitol, and 0.2% (wt/vol) Tween-20) at 25°C in the dark for 30 min. Then the reaction mixtures were analyzed by 4% native polyacrylamide gel. The images were acquired by a fluorescent image analyzer (Amersham Typhoon).

### Molecular docking

The molecular docking study was conducted within the MOE 2019 computational environment. Initial preparation of the protein structure involved the assignment of protonation states followed by energy minimization employing the Amber10:EHT force field. The putative binding site was defined as the largest pocket identified by the Site Finder module. Conformational sampling of the ligand was carried out via the Triangle Matcher algorithm, yielding 30 discrete poses. These poses were subsequently evaluated and ranked according to the London dG scoring function. A subset of the 10 top-ranked conformations was subjected to further scrutiny, with the ultimate selection of the optimal pose predicated on a superior docking score and a comprehensive analysis of its binding mode and residue interactions. Molecular interactions were delineated using LigPlus, and schematic representations were rendered in PyMOL.

### Detection of vancomycin binding to ABC protein using SPR

The CM5 chip was used for detection, and the sensor surface was activated by injecting a mixture of 50 mM N-hydroxysuccinimide and 200 mM 1-ethyl-3-(3-dimethylaminopropyl) carbodiimide for 7 min. The target protein ABC was then diluted to 10 μg/mL using 10 mM acetate buffer (pH 5.5) and immobilized onto the CM5 chip surface at a flow rate of 10 μL/min for a duration of 420 s. The surface was blocked with 1 M ethanolamine (pH 8.5), resulting in a coupling amount of 3107.9 response units. Subsequently, the binding characteristics of vancomycin to the ABC-like protein were preliminarily determined and evaluated in manual mode, with 100 μM set as the highest analytical concentration of vancomycin, and a twofold dilution series set with a total of eight analysis concentrations. The concentration gradient was 0 μM, 1.5625 μM, 3.125 μM, 6.25 μM, 12.5 μM, 25 μM, 50 μM, and 100 μM. The sample analysis was performed at a flow rate of 30 μL/min, with a binding time of 120 s and a dissociation time of 240 s. Finally, the experiment was performed using a multi-cycle kinetics approach, with the analysis time plotted on the *x*-axis and the response value plotted on the *y*-axis. The Biacore T200 evaluation software was used to calculate the equilibrium dissociation constant (Kd) for each sample.

### Zeta potential measurement

*S. aureus* colonies were grown overnight in TSB with shaking at 220 rpm and 37°C, and the bacterial cells were collected by centrifugation (8,000 × *g*, 5 min, 4°C) and washed twice with PBS. The cells were then resuspended in a 1 mM NaCl solution to achieve a bacterial concentration of 6 × 10^10^ CFU/mL. The zeta potential of bacterial cells was determined using a Zeta Potential Analyzer (Malvern Zetasizer Nano ZS90).

### Cytochrome C binding assay

The determination of the whole-cell surface charge was performed as previously described (45) with slight modification. Overnight cultures of XN108 and Δ*ABC-like* mutant strains were diluted in TSB with an initial OD_600_ = 0.05 and allowed to grow until the early stationary phase (OD_600_ = 6). A 4 mL bacterial suspension was pelleted and washed twice with 20 mM MOPS (morpholinepropanesulfonic acid, pH 7). The bacterial pellet was resuspended in 500 μL of MOPS buffer and incubated with 500 μL of cytochrome C (0.5 mg/mL) at room temperature for 30 min. After incubation, the supernatant was collected by centrifugation at 18,000 × *g* for 30 min, and the absorbance at 530 nm (OD_530_) was measured using an ultraviolet spectrophotometer (UV-1900i).

### Vancomycin concentration measurement

The overnight-cultured Staphylococcus aureus strains were transferred to fresh TSB medium (initial OD_600_ = 0.05) and cultured until the OD_600_ reached the mid-logarithmic phase. Vancomycin (40 μg/mL) was then added for a 1 h treatment. The bacterial cells were collected by centrifugation at 10,000 × *g* for 10 min and washed thrice with PBS. The cells were then lysed using a cell disruptor, and the lysate was centrifuged at 10,000 × *g* for 10 min to collect the supernatant. Samples were analyzed using high-performance liquid chromatography (Agilent, HPLC1200) with a C18 column (Dikma, 4.6 × 250 mm, 5 μm) at 30°C using three solvents (A: methanol, B: acetonitrile, C: 0.5% acetic acid). The injection volume was set at 20 μL, with a constant flow rate of 1 mL/min. The elution gradient was programmed as follows: 0 min, 10% A, 5% B, 85% C; 5 min, 10% A, 5% B, 85% C; 15 min, 20% A, 10% B, 70% C; 20 min, 20% A, 8% B, 72% C; 22 min, 20% A, 8% B, 72% C; 25 min, 10% A, 5% B, 85% C; 35 min, 10% A, 5% B, 85% C. Vancomycin quantification was performed using a calibration curve generated by plotting the ion peak area against the corresponding concentration.

### Mouse model

Female BALB/c mice (outbred, immunocompetent, aged 6–8 weeks; purchased from GemPharmatech Co., Ltd.) were utilized to establish a mouse infection model. The mice were housed in individually ventilated cages within a specific-pathogen-free animal facility. To quantify bacterial colonization in the internal organs, a group size of six animals was used. The study followed a randomized design, with blinding maintained throughout the experimental and analytical procedures. *S. aureus* isolates were cultured in TSB and allowed to grow until the OD_600_ reached mid-logarithmic phase. Then, the cells were collected and washed twice with PBS. The mice were inoculated with 200 μL of 0.9% NaCl containing 1 × 10^8^ CFU of each target strain via intraperitoneal injection. Vancomycin was administered intraperitoneally at a daily dose of 32 mg/kg (at 24 h intervals) for four consecutive days. Following euthanasia by cervical dislocation, the organs of the mice were excised and homogenized in PBS. The number of CFU recovered from the homogenized organs was determined by serial dilution on LA plates. For histological analysis, the organs were fixed in a general-purpose tissue fixation solution (Servicebio, G1101) and sent to Hangzhou Powerful Biology for paraffin embedding and H&E staining.

### Statistical analyses

Statistical analyses were performed with a Student’s *t*-test or two-way ANOVA test. Differences with a *P*-value of 0.05 or less were considered significant. Graphing and analysis were performed using GraphPad Prism 10. All experiments were performed in biological triplicates. Statistical details for each experiment can be found in the figure legends.

## Data Availability

RNA sequencing data were deposited in the NCBI database and are publicly available in BioProject (https://www.ncbi.nlm.nih.gov/sra/PRJNA1104410; BioProject accession number: PRJNA1104410).

## SUPPLEMENTAL MATERIAL

Supplemental figures msystems.01630-25-s0001.docx Figures S1 to S3 and raw images.

Source data msystems.01630-25-s0002.xlsx Source data for Fig. S3.

Table S1 msystems.01630-25-s0003.docx Strains and plasmids used in this study.

Table S2 msystems.01630-25-s0004.docx Primers used in this study.

Table S3 msystems.01630-25-s0005.docx Differentially expressed genes (Δ*sarA* vs XN108) from RNA-seq.

Table S4 msystems.01630-25-s0006.docx Assessment of binding affinity between ABC-like and vancomycin.

Table S5 msystems.01630-25-s0007.docx MICs for vancomycin in different *S. aureus* strains.

ASM does not own the copyrights to Supplemental Material that may be linked to, or accessed through, an article. The authors have granted ASM a non-exclusive, world-wide license to publish the Supplemental Material files. Please contact the corresponding author directly for reuse.
