# Supplementary material for: Staphylococcal accessory regulator SarA-mediated modulation of autolysis and surface charge enables Staphylococcus aureus to evade vancomycin killing
Source: mSystems. 2026 Feb 9;11(3):e01630-25. doi: 10.1128/msystems.01630-25 (PMC13011385; doi:10.1128/msystems.01630-25)
Supplement: Supplemental figures — Figures S1 to S3 and raw images. [file msystems.01630-25-s0001.docx]

**SUPPLEMENTARY INFORMATION**


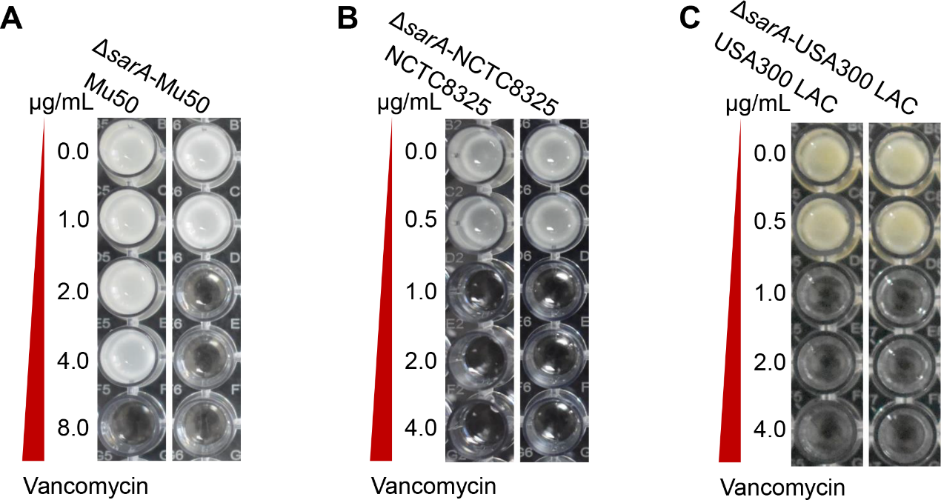


**Fig. S1. Disruption of *sarA* in Mu50 increases the susceptibility of *S. aureus* to vancomycin.** MICs of wild-type *S. aureus* Mu50 and Δ*sarA*-Mu50. All the strains were grown under vancomycin conditions in a 96-well plate with constant shaking at 200 rpm for 48 h at 37°C.


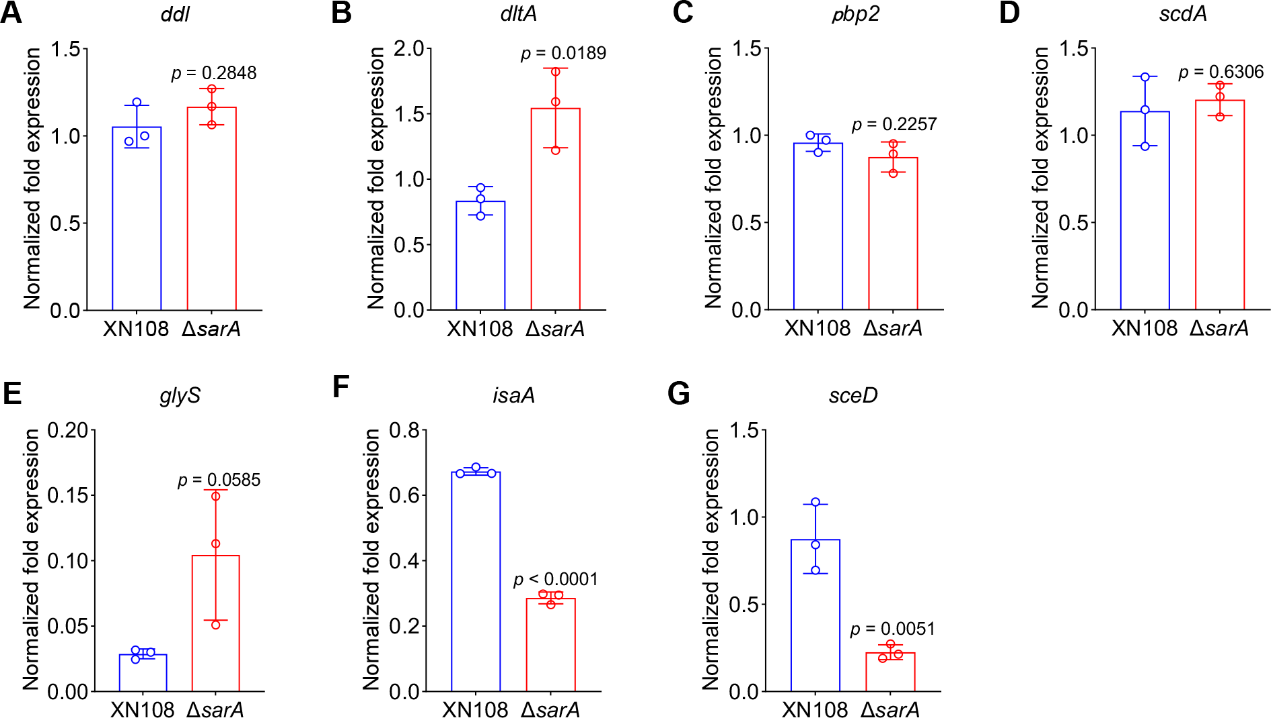


**Fig. S2. Expression of genes related to cell wall synthesis or autolysis in *S. aureus******.*** For (A–G), all experiments were performed in triplicate. The data are represented as the means ± SD. Statistical significance was determined by the two-tailed unpaired Student’s *t*-test.


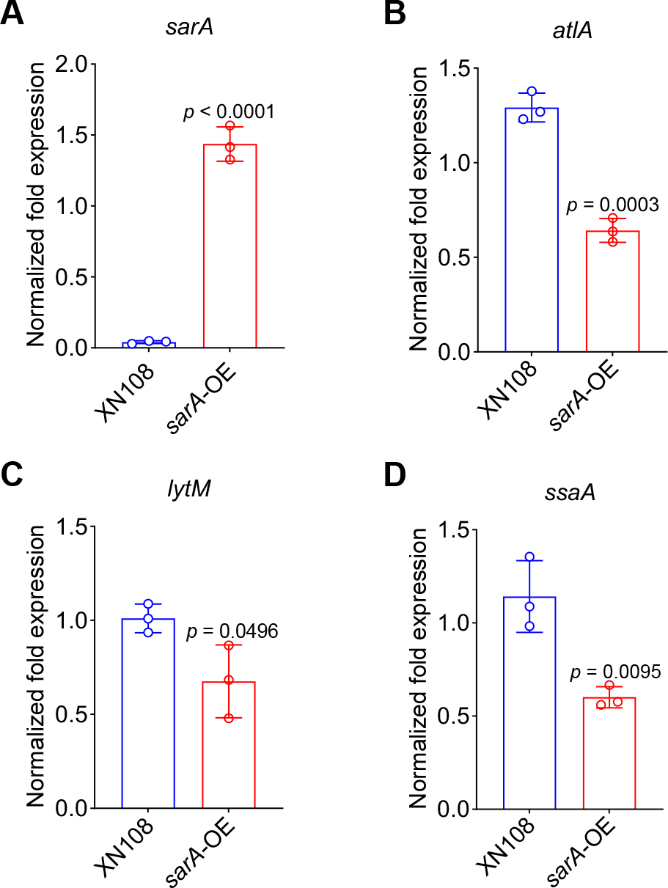


**Fig. S3. The overexpression of the *sarA* gene inhibits the transcription of autolysis-related genes.** For (A–D), all experiments were performed in triplicate. The data are represented as the means ± SD. Statistical significance was determined by the two-tailed unpaired Student’s *t*-test.

**
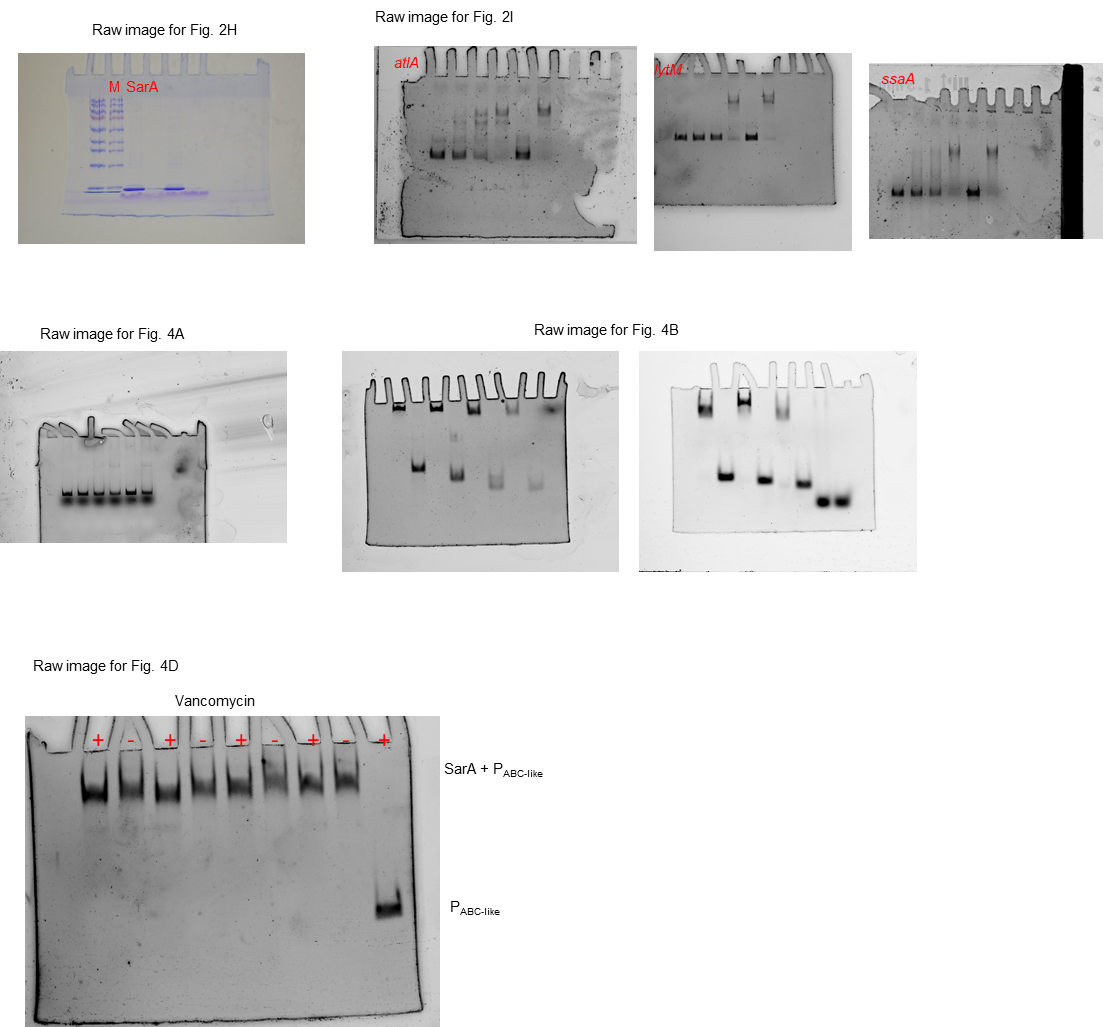
RAW IMAGES**
