# Supplementary material for: Staphylococcal accessory regulator SarA-mediated modulation of autolysis and surface charge enables Staphylococcus aureus to evade vancomycin killing
Source: mSystems. 2026 Feb 9;11(3):e01630-25. doi: 10.1128/msystems.01630-25 (PMC13011385; doi:10.1128/msystems.01630-25)
Supplement: Table S1 — Strains and plasmids used in this study. [file msystems.01630-25-s0003.docx]

**Table S1. Strains and plasmids used in this study.**

| **Strain or plasmid** | **Description** | **Reference or source** |
| --- | --- | --- |
| ***S. aureus* strains** |  |  |
| RN4220 | 8325-4 ^a^r^-^, initial recipient for modification of plasmids which are introduced into *S. aureus* from *E. coli* | NARSA^b^ |
| XN108 | VISA strain, ST239-MRSA-SCC*mec* III | (Rao et al., 2021). |
| Δ*sarA* | Disruption of *sarA* in XN108 | This study |
| Δ*sarA*::*sarA* | Chromosomal complementation of *sarA* mutant | This study |
| Δ*ABC-like* | Disruption of *ABC* in XN108 | This study |
| Δ*sarA*Δ*ABC-like* | Disruption of *sarA* and *ABC* in XN108 | This study |
| Mu50 | VISA, ST5 | NARSA^b^ |
| Mu50Δ*sarA* | Disruption of *sarA* in Mu50 | This study |
| ***E. coli* strains** |  |  |
| DH5α | Clone host strain, F- φ80*lac* ZΔM15 Δ(lacZYA-*arg* F) U169 *end*A1 *rec*A1 *hsd*R17(rk^-^,mk^+^) *sup*E44λ- *thi*-1 *gyr*A96 *rel*A1*pho*A | Yeasen |
| BL21 | F- *omp*T *hsd*SB (rB^-^ mB^-^) *gal* *dcm* (DE3) | TransGen |
| **Plasmids** |  |  |
| pBTs | Shuttle vector, temp sensitive, amp^r^ cm^r^ | This study |
| pBTs-LB-*sarA*-RB | pBTs derivative, for *narGHJI* deletion, amp^r^ cm^r^ | This study |
| pBTs-*sarA*-Com | pBTs derivative, for *sarA* chromosomal complementation, amp^r^ cm^r^ | This study |
| pLI50-*sarA*-OE | *sarA* Overexpression, amp^r^ cm^r^ | This study |
| pBTs-LB-*ABC-like*-RB | pBTs derivative, for *ABC* deletion, amp^r^ cm^r^ | This study |
| PALC-P*_sarA_*::GFP | *sarA* promoter activity assay, amp^r^ cm^r^ | This study |

^a^r^-^, restriction system negative; kan^r^, kanamycin resistant; amp^r^, ampicillin resistant; cm^r^, chloramphenicol resistant; str^r^, streptomycin resistant.

^b^NARSA, Network on antimicrobial resistance in *S. aureus*.
