# Supplementary material for: Staphylococcal accessory regulator SarA-mediated modulation of autolysis and surface charge enables Staphylococcus aureus to evade vancomycin killing
Source: mSystems. 2026 Feb 9;11(3):e01630-25. doi: 10.1128/msystems.01630-25 (PMC13011385; doi:10.1128/msystems.01630-25)
Supplement: Table S2 — Primers used in this study. [file msystems.01630-25-s0004.docx]

**Table S2.** **Primers used in this study.**

| **Primer name** | **Sequence (5′to 3′)** | **Comments** |
| --- | --- | --- |
| *sarA*-LB-F | GGGGTACCGATAGTCAACTCATTCTTAAGAC | *sarA* disruption in XN108 |
| *sarA*-LB-R | TTTGCTTCAGTGATTCGGTTTAAAACCTCCCTATTTG | *sarA* disruption in XN108 |
| *sarA*-RB-F | CGAATCACTGAAGCAAA | *sarA* disruption in XN108 |
| *sarA*-RB-R | CGGGATCCGGACATGATTTAGGTGGG | *sarA* disruption in XN108 |
| *sarA*-LB(com)-F | GCGGAATTCGAGCTCGGTACCGATAGTCAACTCATTCTTAAGAC | *sarA* chromosomal complementation |
| *SarA*-LB(com)-R | TGACAACAACTCAAAGCAATCATTGATTTTTGTAATTGCC | *sarA* chromosomal complementation |
| *sarA*-RB(com)-F | ATTGCTTTGAGTTGTTGTCAATGGTCACTTATGCTGACAA | *sarA* chromosomal complementation |
| *sarA*-RB(com)-R | CTTGCATGCCTGCAGGTCGACGGACATGATTTAGGTGGG | *sarA* chromosomal complementation |
| *sarA*^50^-LB-F | GCGGAATTCGAGCTCGGTACCGATAGTCAACTCATTCTTAAGA | *sarA* disruption in Mu50 |
| *sarA*^50^-LB-R | TTCGTTGTTTGCTTCAGTGATTCGGTTTAAAACCTCCCTATTTG | *sarA* disruption in Mu50 |
| *sarA*^50^-RB-F | GCATCAAATAGGGAGGTTTTAAACCGAATCACTGAAGCAAA | *sarA* disruption in Mu50 |
| *sarA*^50^-RB-R | CTTGCATGCCTGCAGGTCGACGGACATGATTTAGGTGGG | *sarA* disruption in Mu50 |
| *ABC*-LB-F | GCGGAATTCGAGCTCGGTACCTTACTATTCCCAATCCCGTT | *ABC* disruption |
| *ABC*-LB-R | AAGTTCCCATTTTATGCTTGGTCCCCATATCACTATCTCCTTTG | *ABC* disruption |
| *ABC*-RB-F | ATAGCAAAGGAGATAGTGATATGGGGACCAAGCATAAAATGGGA | *ABC* disruption |
| *ABC*-RB-R | CTTGCATGCCTGCAGGTCGACGGTATTAGGAACCGTTGCAG | *ABC* disruption |
| *pta*-F | AAAGCGCCAGGTGCTAAATTAC | Internal reference in RT-qPCR analysis |
| *pta*-R | CTGGACCAACTGCATCATATCC | Internal reference in RT-qPCR analysis |
| *sarA*ex-F | ATGGTCACTTATGCTGACAA | RT-qPCR analysis of *sarA* |
| *sarA*ex-R | GGTTGTTTGTAGTTTAAATG | RT-qPCR analysis of *sarA* |
| *sarA*-OE-F | AGCTCGGTACCCGGGGATCCGACTAAACCAAATGCTAACCCAG | Overexpression of *sarA* |
| *sarA*-OE-R | AGGTCGACTCTAGAGGATCCTTATTATAGTTCAATTTCGTTG | Overexpression of *sarA* |
| *ABC*ex-F | GCAATATCGGCGCATTGATT | RT-qPCR analysis of *ABC* |
| *ABC*ex-R | GCCATTCATTGGTTCATCTA | RT-qPCR analysis of *ABC* |
| *atlA*-F | CAGCACCAACGGATTACTTA | RT-qPCR analysis of *atlA* |
| *atlA*-R | CATACTCAGCACTGTCTGGT | RT-qPCR analysis of *atlA* |
| *lytM*-F | ACATTCGTAGATGCACAAGG | RT-qPCR analysis of *lytM* |
| *lytM*-R | TAATAGCTCGCGTCTGGACC | RT-qPCR analysis of *lytM* |
| *ssaA*-F | CGCATCATGATGCACAAGCT | RT-qPCR analysis of *ssaA* |
| *ssaA*-R | TTGTGGAGAAGTGTTTGACG | RT-qPCR analysis of *ssaA* |
| P*_ABC_*-F | GCGCATTTTAATAAATGTAG | EMSA |
| P*_ABC_-*R | ATCACTATCTCCTTTGCTAT | Labeling with 6-FAM, EMSA |
| P*_ABC_*_136_-F | CTCGTATTCATATTTATG | EMSA |
| P*_ABC_*_93_-F | TCCATATTTTTATAACACTTGC | EMSA |
| P*_ABC_*_81_-F | TAACACTTGCTATGTGATAA | EMSA |
| P*_ABC_*_70_-F | ATGTGATAAAATTAATTTTATATATAAAATCTTAAGATTCAG | EMSA |
| P*_ABC_*_60_-F | ATTAATTTTATATATAAAATCTTAAGATTCAG | EMSA |
| P*_ABC_*_50_-F | TATATAAAATCTTAAGATTCAG | EMSA |
| P*_ABC_*_20_-F | ATAGCAAAGGAGATAGTGAT | EMSA |
| P*_atlA_*-F | ACTTTAAAATGAGAGGTAAT | EMSA |
| P*_atlA_*-R | TCTATTTATTACTCCTAGC | Labeling with 6-FAM, EMSA |
| P*_lytM_*-F | CGATTGAAAATGGAATTTGG | EMSA |
| P*_lytM_*-R | GTATAAAACATCCTCCATTA | Labeling with 6-FAM, EMSA |
| P*_ssaA_*-F | AATAATTCCTCCTTCTACCGTCG | EMSA |
| P*_ssaA_*-R | AGGGACTCCTCCTTAAAATT | Labeling with 6-FAM, EMSA |
| P*_sarA-Ac_*-F | AAACGACGGCCAGTGAATTCCCTTGTATTGTCGATTAAATTAAGG | Activity detection of *sarA* promoter |
| P*_sarA-Ac_*-R | ACCATGGTGGCGACGAATTCGTTTAAAACCTCCCTATTTGATGC | Activity detection of *sarA* promoter |
| *ABC*-P-F | AAACGACGGCCAGTGAATTCGCGCATTTTAATAAATGTAG | Expression of ABC protein in *S. aureus* |
| *ABC*-ORF-R | AAAAGCTTGCATGCCTGCAGTTAGTGATGATGATGATGATGTGCTTGGTCCTCCCTTTC | Expression of ABC protein in *S. aureus* |
| PET-*ABC*-F | CTTTAAGAAGGAGATATACCATGGATGTTTTAACAATAGAAC | Expression of ABC protein in *E.coli* |
| PET-*ABC*-R | GTGGTGGTGGTGGTGGTGCTCGAGTGCTTGGTCCTCCCTTTCTA | Expression of ABC protein in *E.coli* |
| PET-*sarA*-F | CTTTAAGAAGGAGATATACCATGGCAATTACAAAAATCAATGATTGC | Expression of SarA protein |
| PET-*sarA*-R | GTGGTGGTGGTGGTGGTGCTCGAGTAGTTCAATTTCGTTGTTTGC | Expression of SarA protein |
|  |  |  |
