# Supplementary material for: Staphylococcal accessory regulator SarA-mediated modulation of autolysis and surface charge enables Staphylococcus aureus to evade vancomycin killing
Source: mSystems. 2026 Feb 9;11(3):e01630-25. doi: 10.1128/msystems.01630-25 (PMC13011385; doi:10.1128/msystems.01630-25)
Supplement: Table S3 — Differentially expressed genes (ΔsarA vs XN108) from RNA-seq. [file msystems.01630-25-s0005.docx]

**Table S3. Differentially expressed genes (Δ*sarA* vs XN108) from RNA-seq.** RNA-seq analysis was performed to compare the significantly differentially expressed genes between sarA gene-knockout mutant and XN108. The Gene ID and Gene name information in the table was obtained from https://www.ncbi.nlm.nih.gov/nuccore/CP007447.1 and the RNA-seq raw data were deposited in the NCBI database (https://www.ncbi.nlm.nih.gov/sra/PRJNA1104410, BioProject accession number: PRJNA1104410).

| **Gene name** | **Gene description** | | **XN108 (average of 3 replicates)** | **Δ*sarA* (average of 3 replicates)** | **Log2FC(Δ*sarA*/XN108)** | **P-value** | **P-adjust** | **Significant** | **Regulate** |
| --- | --- | --- | --- | --- | --- | --- | --- | --- | --- |
| SAXN108_0262 | Esat-6/Esx Family Secreted Protein Esxa/Yuke | 1006.79 | | 36593.08667 | 5.353054213 | 0 | 0 | Yes | Up |
| SAXN108_0860 | Thermonuclease Precursor | 4.3 | | 2771.2 | 9.536941564 | 0 | 0 | Yes | Up |
| SAXN108_0119 | Superoxide Dismutase | 27.13333333 | | 2518.21 | 6.72215404 | 0 | 0 | Yes | Up |
| SAXN108_2009 | Staphopain A Precursor | 1.15 | | 760.3066667 | 9.543074579 | 0 | 0 | Yes | Up |
| SAXN108_0096 | Immunoglobulin G Binding Protein A | 1.93 | | 2694.906667 | 10.62536669 | 0 | 0 | Yes | Up |
| SAXN108_2935 | Putative Capsule Synthesis Protein | 8.05 | | 382.9066667 | 5.755774304 | 7.91E-294 | 3.91E-291 | Yes | Up |
| SAXN108_2455 | Hyaluronate Lyase | 3.82 | | 175.4066667 | 5.703389227 | 5.73E-278 | 2.43E-275 | Yes | Up |
| SAXN108_1153 | Fibrinogen-Binding Protein | 17.60333333 | | 547.7066667 | 5.133103409 | 6.77E-232 | 2.51E-229 | Yes | Up |
| SAXN108_0129 | Phosphonate Abc Transporter Phosphate-Binding Periplasmic Component | 1.436666667 | | 156.0666667 | 6.968657802 | 6.13E-221 | 2.02E-218 | Yes | Up |
| SAXN108_2045 | Staphylococcal Complement Inhibitor Scin | 221.6466667 | | 4357.8 | 4.479708792 | 9.64E-208 | 2.86E-205 | Yes | Up |
| SAXN108_0975 | Surface Protein | 227.7133333 | | 5225.226667 | 4.702452219 | 4.05E-206 | 1.09E-203 | Yes | Up |
| SAXN108_2673 | Igg-Binding Protein Sbi | 8.373333333 | | 562.58 | 6.248322521 | 2.11E-204 | 5.22E-202 | Yes | Up |
| SAXN108_1049 | Putative Atl Autolysin Transcription Regulator | 19.52 | | 912.1233333 | 5.734383629 | 1.39E-199 | 3.17E-197 | Yes | Up |
| SAXN108_2936 | Putative Capsule Synthesis Protein | 3.986666667 | | 275.5166667 | 6.29057494 | 7.88E-195 | 1.67E-192 | Yes | Up |
| SAXN108_2666 | Femab Family Protein | 5.38 | | 135.5133333 | 4.836924193 | 9.13E-179 | 1.81E-176 | Yes | Up |
| SAXN108_2764 | Hypothetical Protein | 1.876666667 | | 185.9466667 | 6.789911542 | 3.37E-171 | 6.25E-169 | Yes | Up |
| SAXN108_0261 | Hypothetical Protein | 10.79 | | 173.1333333 | 4.185303538 | 4.44E-167 | 7.75E-165 | Yes | Up |
| SAXN108_2047 | Staphylokinase | 6.56 | | 147.32 | 4.675107502 | 8.46E-163 | 1.39E-160 | Yes | Up |
| SAXN108_2979 | Hypothetical Protein | 7.986666667 | | 135.36 | 4.264847652 | 7.49E-153 | 1.17E-150 | Yes | Up |
| SAXN108_0263 | Putative Secretion Accessory Protein Esaa/Yueb | 2.143333333 | | 43.4 | 4.518519146 | 2.03E-144 | 3.01E-142 | Yes | Up |
| SAXN108_1032 | Glycosyl Transferase%2C Group 1 Family Protein | 2.216666667 | | 66.54333333 | 5.112011604 | 1.52E-143 | 2.15E-141 | Yes | Up |
| SAXN108_2763 | Abc Transporter Atp-Binding Protein | 0.996666667 | | 145.5966667 | 7.396122484 | 1.25E-140 | 1.68E-138 | Yes | Up |
| SAXN108_0618 | Serine-Aspartate Repeat-Containing Protein D | 2.146666667 | | 115.7766667 | 5.93466924 | 1.01E-139 | 1.30E-137 | Yes | Up |
| novel0001 | Protein A, Partial [Staphylococcus Aureus] | 2.85 | | 2140.976667 | 9.717054619 | 2.96E-139 | 3.66E-137 | Yes | Up |
| SAXN108_2010 | Hypothetical Protein | 4.696666667 | | 456.6466667 | 6.821713263 | 6.09E-138 | 7.23E-136 | Yes | Up |
| SAXN108_2926 | Serine-Rich Adhesin For Platelets | 20.62333333 | | 200.1433333 | 3.462436976 | 3.09E-136 | 3.53E-134 | Yes | Up |
| SAXN108_2109 | Leukocidin Lukf-Pv | 1.696666667 | | 60.75 | 5.364951464 | 7.79E-133 | 8.56E-131 | Yes | Up |
| SAXN108_0128 | Phosphonate Abc Transporter Atp-Binding Protein | 1.1 | | 52.78 | 5.778933424 | 6.06E-132 | 6.41E-130 | Yes | Up |
| SAXN108_2887 | Putative Transporter Protein | 4.58 | | 63.10333333 | 3.973094175 | 3.84E-127 | 3.93E-125 | Yes | Up |
| SAXN108_2908 | Zinc Metalloproteinase Precursor | 22.66 | | 351.9166667 | 4.14472242 | 5.41E-127 | 5.35E-125 | Yes | Up |
| SAXN108_2129 | Accessory Gene Regulator B | 101.5266667 | | 671.26 | 2.907658425 | 6.09E-120 | 5.83E-118 | Yes | Up |
| SAXN108_2677 | Gamma-Hemolysin Component B | 3.23 | | 87.94 | 4.950028598 | 6.65E-117 | 6.16E-115 | Yes | Up |
| SAXN108_0629 | Had Family Hydrolase | 8.55 | | 294.2866667 | 5.2943764 | 8.81E-117 | 7.92E-115 | Yes | Up |
| SAXN108_0114 | Nad Dependent Epimerase/Dehydratase Family Protein | 0.39 | | 54.38 | 7.259024311 | 1.12E-111 | 9.80E-110 | Yes | Up |
| SAXN108_1460 | Alanine Dehydrogenase | 1.18 | | 104.34 | 6.656433832 | 1.02E-108 | 8.66E-107 | Yes | Up |
| SAXN108_2955 | Hypothetical Protein | 2.903333333 | | 57.68666667 | 4.502860666 | 5.56E-105 | 4.58E-103 | Yes | Up |
| SAXN108_1150 | Hypothetical Protein | 13.85666667 | | 155.5066667 | 3.666015291 | 2.01E-103 | 1.61E-101 | Yes | Up |
| SAXN108_0126 | Binding-Protein-Dependent Transport Systemsmembrane Component | 2.696666667 | | 58.58666667 | 4.632926059 | 3.81E-101 | 2.97E-99 | Yes | Up |
| SAXN108_2676 | Gamma-Hemolysin Component C | 1.073333333 | | 88.18 | 6.506669867 | 4.75E-100 | 3.61E-98 | Yes | Up |
| SAXN108_2773 | D-Lactate Dehydrogenase | 363.6 | | 2456.08 | 2.948388675 | 1.22E-99 | 9.03E-98 | Yes | Up |
| SAXN108_1717 | Hypothetical Protein | 3.696666667 | | 40.53333333 | 3.648681423 | 1.83E-98 | 1.32E-96 | Yes | Up |
| SAXN108_0134 | Alcohol Dehydrogenase%3B Acetaldehyde Dehydrogenase | 6.266666667 | | 98.23666667 | 4.163441249 | 1.87E-98 | 1.32E-96 | Yes | Up |
| SAXN108_1459 | Threonine Dehydratase%2C Catabolic | 1.073333333 | | 120.5133333 | 7.002610613 | 1.13E-95 | 7.82E-94 | Yes | Up |
| SAXN108_2980 | Hypothetical Protein | 6.173333333 | | 101.69 | 4.219439263 | 5.42E-94 | 3.66E-92 | Yes | Up |
| SAXN108_2901 | Fibrinogen And Keratin-10 Binding Surfaceanchored Protein | 16.79666667 | | 99.27 | 2.748186248 | 1.29E-93 | 8.47E-92 | Yes | Up |
| SAXN108_1149 | Hypothetical Protein | 81.44 | | 2419.296667 | 5.068253735 | 1.58E-93 | 1.02E-91 | Yes | Up |
| SAXN108_1458 | Amino Acid Permease | 2.95 | | 120.1533333 | 5.537273083 | 4.28E-93 | 2.70E-91 | Yes | Up |
| SAXN108_0699 | Abc Transporter Atp-Binding Protein | 57.48333333 | | 308.94 | 2.616893797 | 4.42E-92 | 2.73E-90 | Yes | Up |
| SAXN108_0291 | Hypothetical Protein | 90.13333333 | | 740.8 | 3.228355533 | 3.80E-91 | 2.30E-89 | Yes | Up |
| SAXN108_1154 | Fibrinogen-Binding Protein | 5.626666667 | | 180.81 | 5.206490437 | 1.65E-90 | 9.79E-89 | Yes | Up |
| SAXN108_0127 | Binding-Protein-Dependent Transport Systemmembrane Component | 0.91 | | 32.47666667 | 5.335234787 | 8.04E-86 | 4.68E-84 | Yes | Up |
| SAXN108_2110 | Leukocidin Luks-Pv | 1.49 | | 61.19666667 | 5.514029217 | 5.68E-85 | 3.24E-83 | Yes | Up |
| SAXN108_2925 | Adhesin | 28.87666667 | | 268.8066667 | 3.406444532 | 1.75E-82 | 9.80E-81 | Yes | Up |
| SAXN108_1046 | Glutamyl Endopeptidase Precursor%2C Serine Proteinase Sspa | 9.676666667 | | 85.07 | 3.313562055 | 1.05E-80 | 5.75E-79 | Yes | Up |
| SAXN108_2128 | Delta-Hemolysin Precursor | 220.61 | | 5937.04 | 4.929571985 | 1.82E-80 | 9.79E-79 | Yes | Up |
| SAXN108_0260 | Hypothetical Protein | 23.89666667 | | 232.5866667 | 3.463722417 | 6.86E-80 | 3.64E-78 | Yes | Up |
| SAXN108_1828 | Hypothetical Protein | 13.52 | | 81.25666667 | 2.776740327 | 1.04E-79 | 5.39E-78 | Yes | Up |
| SAXN108_0970 | Lysr Family Regulatory Protein | 0.663333333 | | 56.09333333 | 6.619066766 | 4.01E-79 | 2.05E-77 | Yes | Up |
| SAXN108_0180 | Type I Restriction-Modification System%2Crestriction Subunit R | 9.613333333 | | 43.61 | 2.372025719 | 3.01E-78 | 1.51E-76 | Yes | Up |
| SAXN108_2862 | Hypothetical Protein | 9.243333333 | | 95.88333333 | 3.558961713 | 4.20E-77 | 2.07E-75 | Yes | Up |
| SAXN108_2041 | Mhc Class Ii-Like Protein | 3.723333333 | | 29.09666667 | 3.152656048 | 5.05E-77 | 2.45E-75 | Yes | Up |
| SAXN108_2050 | Enterotoxin Type A | 88.84666667 | | 385.49 | 2.309378977 | 1.53E-76 | 7.30E-75 | Yes | Up |
| SAXN108_0214 | Hypothetical Protein | 10.21 | | 54.12666667 | 2.59396833 | 1.79E-76 | 8.43E-75 | Yes | Up |
| SAXN108_0305 | Triacylglycerol Lipase | 2.843333333 | | 40.39666667 | 4.00095363 | 1.37E-75 | 6.34E-74 | Yes | Up |
| SAXN108_0868 | Phosphoglycerate Mutase Family | 33.47 | | 244.44 | 3.054094788 | 1.40E-75 | 6.41E-74 | Yes | Up |
| SAXN108_2886 | Anaerobic Ribonucleoside-Triphosphate Reductase | 24.46 | | 112.83 | 2.393666386 | 4.74E-74 | 2.13E-72 | Yes | Up |
| SAXN108_2131 | Accessory Regulator Protein C | 35.94666667 | | 217.2233333 | 2.787333692 | 1.32E-71 | 5.84E-70 | Yes | Up |
| SAXN108_0697 | Abc Transporter Permease | 103.8333333 | | 470.3533333 | 2.372484584 | 1.89E-70 | 8.25E-69 | Yes | Up |
| SAXN108_0294 | Hypothetical Protein | 42.96 | | 172.57 | 2.196110753 | 8.34E-69 | 3.58E-67 | Yes | Up |
| SAXN108_0025 | Putative Lpxtg Surface 5'-Nucleotidase | 6.463333333 | | 34.85333333 | 2.61822619 | 1.02E-68 | 4.32E-67 | Yes | Up |
| SAXN108_0086 | 1-Phosphatidylinositol Phosphodiesterase | 0.673333333 | | 18.07666667 | 4.937365645 | 5.05E-68 | 2.11E-66 | Yes | Up |
| SAXN108_0671 | Alcohol Dehydrogenase | 106.02 | | 1136.25 | 3.614934765 | 1.40E-67 | 5.79E-66 | Yes | Up |
| SAXN108_2228 | Hypothetical Protein | 40.2 | | 4.256666667 | -3.051143745 | 2.89E-67 | 1.17E-65 | Yes | Down |
| SAXN108_1856 | Gamma-Hemolysin Component B | 1.03 | | 24.36666667 | 4.726953841 | 7.98E-66 | 3.20E-64 | Yes | Up |
| SAXN108_1811 | Transaldolase | 252.0066667 | | 1141.206667 | 2.372109222 | 2.81E-64 | 1.11E-62 | Yes | Up |
| SAXN108_0266 | Putative Secretion System Component Essb/Yukc | 1.263333333 | | 17.33 | 3.97098791 | 5.17E-61 | 2.02E-59 | Yes | Up |
| SAXN108_0116 | Galactosyl Transferase | 0.58 | | 15.67333333 | 4.960772762 | 7.90E-61 | 3.04E-59 | Yes | Up |
| SAXN108_1457 | Putative Transporter | 2.45 | | 41.18666667 | 4.259097733 | 6.99E-60 | 2.66E-58 | Yes | Up |
| SAXN108_0619 | Serine-Aspartate Repeat-Containing Protein E | 27.18 | | 185.8733333 | 2.953159657 | 1.39E-59 | 5.22E-58 | Yes | Up |
| SAXN108_0698 | Abc Transporter Permease | 24.36333333 | | 124.2766667 | 2.535291878 | 8.86E-59 | 3.28E-57 | Yes | Up |
| SAXN108_2642 | Nitrate/Nitrite Transporter | 939.9466667 | | 132.09 | -2.635346041 | 2.05E-58 | 7.51E-57 | Yes | Down |
| SAXN108_2256 | Phage Protein | 214.5366667 | | 34 | -2.474305942 | 3.07E-58 | 1.11E-56 | Yes | Down |
| SAXN108_0231 | Putative Zinc-Binding Dehydrogenase | 17.65666667 | | 88.92666667 | 2.52298178 | 7.02E-58 | 2.51E-56 | Yes | Up |
| SAXN108_0232 | Putative Cdp-Glycerol:Poly(Glycerophosphate) Glycerophosphotransferase | 51.21666667 | | 225.65 | 2.333031398 | 9.21E-58 | 3.25E-56 | Yes | Up |
| SAXN108_1045 | Cysteine Protease Sspb | 5.11 | | 32.53 | 2.849695223 | 1.02E-57 | 3.55E-56 | Yes | Up |
| SAXN108_2516 | Hth-Type Transcriptional Regulator Sarv | 33.19333333 | | 176.5466667 | 2.592121045 | 2.56E-57 | 8.82E-56 | Yes | Up |
| SAXN108_2593 | Hypothetical Protein | 87.48333333 | | 292.9 | 1.932396465 | 3.75E-57 | 1.28E-55 | Yes | Up |
| SAXN108_1329 | Hypothetical Protein | 10.34333333 | | 126.1 | 3.765118361 | 4.43E-56 | 1.49E-54 | Yes | Up |
| SAXN108_2871 | L-Lactate Dehydrogenase 2 | 99.46 | | 474.9933333 | 2.453748923 | 1.50E-55 | 5.00E-54 | Yes | Up |
| SAXN108_2268 | Hypothetical Protein | 32.75 | | 2.953333333 | -3.283143447 | 5.92E-55 | 1.95E-53 | Yes | Down |
| SAXN108_0130 | Hypothetical Protein | 1.396666667 | | 16.52333333 | 3.744687112 | 1.48E-54 | 4.83E-53 | Yes | Up |
| SAXN108_2258 | Hypothetical Protein | 34.38333333 | | 5.25 | -2.525727942 | 4.14E-53 | 1.33E-51 | Yes | Down |
| SAXN108_2262 | Hypothetical Protein | 205.8833333 | | 28.4 | -2.674720761 | 9.05E-53 | 2.89E-51 | Yes | Down |
| SAXN108_2939 | Glucosaminyltransferase | 0.86 | | 27.3 | 5.1798729 | 1.21E-52 | 3.83E-51 | Yes | Up |
| SAXN108_0257 | Abc Transporter Atp-Binding Protein | 11.96666667 | | 69.72333333 | 2.73065637 | 3.45E-52 | 1.08E-50 | Yes | Up |
| SAXN108_2132 | Accessory Gene Regulator Protein A | 26.01 | | 134.6133333 | 2.56078602 | 4.37E-52 | 1.35E-50 | Yes | Up |
| SAXN108_2272 | Hypothetical Protein | 38.77 | | 5.42 | -2.653316562 | 4.79E-52 | 1.46E-50 | Yes | Down |
| SAXN108_0259 | Hypothetical Protein | 3.846666667 | | 21.87666667 | 2.698808326 | 1.06E-51 | 3.22E-50 | Yes | Up |
| SAXN108_2721 | Oligopeptide Transporter Putative Substratebinding Domain | 6.506666667 | | 43.24666667 | 2.918678943 | 1.08E-51 | 3.23E-50 | Yes | Up |
| SAXN108_1026 | Hypothetical Protein | 76.66333333 | | 697.44 | 3.375573488 | 2.14E-51 | 6.35E-50 | Yes | Up |
| SAXN108_2260 | Hypothetical Protein | 111.85 | | 11.32666667 | -3.12705774 | 3.33E-51 | 9.79E-50 | Yes | Down |
| SAXN108_0264 | Putative Secretion System Component Essa | 4.583333333 | | 102.5233333 | 4.631855811 | 6.67E-50 | 1.94E-48 | Yes | Up |
| SAXN108_2923 | Hypothetical Protein | 2.423333333 | | 13.47333333 | 2.660127248 | 8.91E-49 | 2.57E-47 | Yes | Up |
| SAXN108_2269 | Hypothetical Protein | 162.2733333 | | 25.66666667 | -2.475187453 | 1.86E-48 | 5.31E-47 | Yes | Down |
| SAXN108_0244 | Nitric-Oxide Reductase | 1.173333333 | | 9.916666667 | 3.272237692 | 3.81E-48 | 1.08E-46 | Yes | Up |
| SAXN108_0117 | Hypothetical Protein | 1.48 | | 14.21 | 3.471571004 | 3.90E-48 | 1.09E-46 | Yes | Up |
| SAXN108_1168 | Hypothetical Protein | 44.05333333 | | 185.99 | 2.264756656 | 1.54E-47 | 4.28E-46 | Yes | Up |
| SAXN108_2978 | Hypothetical Protein | 3.973333333 | | 47.31 | 3.734243457 | 4.73E-47 | 1.30E-45 | Yes | Up |
| SAXN108_0258 | Hypothetical Protein | 3.68 | | 23.24 | 2.842388979 | 2.45E-46 | 6.67E-45 | Yes | Up |
| SAXN108_2115 | Potassium Transporter Ktrb | 2.903333333 | | 29.51666667 | 3.547785349 | 4.95E-46 | 1.33E-44 | Yes | Up |
| SAXN108_2117 | Na+-Transporting Atp Synthase | 42.21333333 | | 280.09 | 2.885112337 | 6.34E-46 | 1.69E-44 | Yes | Up |
| SAXN108_2595 | Putative Permease | 8.19 | | 55.13666667 | 2.944399638 | 8.23E-45 | 2.18E-43 | Yes | Up |
| SAXN108_1022 | Competence Transcription Factor | 3.526666667 | | 33.53666667 | 3.433180343 | 1.22E-44 | 3.21E-43 | Yes | Up |
| SAXN108_2705 | Amino Acid Transporter | 55.91 | | 11.68333333 | -2.071446065 | 4.51E-42 | 1.17E-40 | Yes | Down |
| SAXN108_2341 | Hypothetical Protein | 1081.936667 | | 56.79333333 | -4.080116578 | 9.30E-42 | 2.40E-40 | Yes | Down |
| SAXN108_2969 | Hypothetical Protein | 42.24 | | 142.52 | 1.948846745 | 1.02E-41 | 2.62E-40 | Yes | Up |
| SAXN108_0267 | Ftsk/Spoiiie Family Protein%2C Putative Secretion System Component Essc/Yuka | 1.053333333 | | 13.42666667 | 3.849989539 | 1.05E-41 | 2.65E-40 | Yes | Up |
|  |  | 30918.13333 | | 6533.84 | -2.102633752 | 2.89E-40 | 7.27E-39 | Yes | Down |
| SAXN108_1212 | Hypothetical Protein | 15.77333333 | | 102.1566667 | 2.883155129 | 2.76E-39 | 6.88E-38 | Yes | Up |
| SAXN108_1599 | Serine Protease | 85.01666667 | | 218.7966667 | 1.548247642 | 1.17E-38 | 2.90E-37 | Yes | Up |
| SAXN108_2225 | Phage Protein | 14.76333333 | | 1.356666667 | -3.259315347 | 2.03E-38 | 4.97E-37 | Yes | Down |
| SAXN108_2940 | Intercellular Adhesion Protein B | 1.296666667 | | 13.91666667 | 3.621528062 | 1.05E-37 | 2.55E-36 | Yes | Up |
| SAXN108_1827 | Hypothetical Protein | 25.91333333 | | 160.3333333 | 2.809333881 | 1.10E-37 | 2.66E-36 | Yes | Up |
| SAXN108_1857 | Gamma-Hemolysin Component A | 0.203333333 | | 12.90333333 | 6.193439457 | 1.28E-37 | 3.06E-36 | Yes | Up |
| SAXN108_1511 | Hypothetical Protein | 10.72 | | 30.54 | 1.698203109 | 2.00E-37 | 4.75E-36 | Yes | Up |
| SAXN108_2273 | Hypothetical Protein | 16.43 | | 3.593333333 | -2.007906294 | 9.67E-37 | 2.28E-35 | Yes | Down |
| SAXN108_0230 | 2-C-Methyl-D-Erythritol 4-Phosphate | 67.78333333 | | 285.9166667 | 2.261544353 | 1.16E-36 | 2.70E-35 | Yes | Up |
| SAXN108_2634 | Hypothetical Protein | 150.4966667 | | 26.55666667 | -2.319477143 | 1.38E-36 | 3.20E-35 | Yes | Down |
| SAXN108_0723 | Hypothetical Protein | 13.88333333 | | 108.3333333 | 3.166167604 | 2.60E-36 | 5.98E-35 | Yes | Up |
| SAXN108_0093 | Putative Myosin-Crossreactive Antigen | 85.24333333 | | 277.63 | 1.900646097 | 5.92E-36 | 1.35E-34 | Yes | Up |
| SAXN108_2254 | Phage Dna-Binding Protein | 230.3633333 | | 35.07666667 | -2.540917286 | 7.02E-36 | 1.59E-34 | Yes | Down |
| SAXN108_2404 | Lpxtg Surface-Anchored Protein | 1.34 | | 4.316666667 | 1.875977669 | 8.96E-36 | 2.01E-34 | Yes | Up |
| SAXN108_2459 | Alpha-Acetolactate Decarboxylase | 176.1333333 | | 44.29333333 | -1.799497009 | 6.62E-35 | 1.48E-33 | Yes | Down |
| SAXN108_2229 | Hypothetical Protein | 26.73 | | 4.146666667 | -2.502568558 | 1.70E-34 | 3.76E-33 | Yes | Down |
| SAXN108_2130 | Accessory Gene Regulator D Pheromone Precursor%2C Type Iii | 28.79333333 | | 174.6566667 | 2.760654525 | 3.94E-34 | 8.66E-33 | Yes | Up |
| SAXN108_2885 | Putative Anaerobic Ribonucleotide Reductase Activating Protein | 20.56666667 | | 86.86666667 | 2.268826601 | 7.41E-34 | 1.62E-32 | Yes | Up |
| SAXN108_2263 | Hypothetical Protein | 50.54333333 | | 6.843333333 | -2.699058425 | 1.22E-32 | 2.64E-31 | Yes | Down |
| SAXN108_2646 | Oxygen Regulatory Protein Nrec | 453.1933333 | | 135.4333333 | -1.55743392 | 1.42E-32 | 3.06E-31 | Yes | Down |
| SAXN108_2259 | Hypothetical Protein | 26.08666667 | | 3.98 | -2.522675399 | 1.78E-32 | 3.79E-31 | Yes | Down |
| SAXN108_1031 | Hypothetical Protein | 2.216666667 | | 70.36333333 | 5.062175274 | 7.96E-32 | 1.69E-30 | Yes | Up |
| SAXN108_2966 | Collagen Adhesin | 241.61 | | 1415.503333 | 2.725816817 | 9.07E-32 | 1.91E-30 | Yes | Up |
| SAXN108_2223 | Phage Putative Phosphoesterase | 12.57333333 | | 2.72 | -2.020494005 | 2.22E-31 | 4.63E-30 | Yes | Down |
| SAXN108_2261 | Hypothetical Protein | 210.7266667 | | 25.96 | -2.85118518 | 2.49E-31 | 5.17E-30 | Yes | Down |
| SAXN108_1855 | Lantibiotic Protein | 64.48333333 | | 453.67 | 2.97410627 | 2.94E-31 | 6.05E-30 | Yes | Up |
| SAXN108_0734 | Arac Family Transcriptional Regulator | 5.933333333 | | 24.48 | 2.237236385 | 3.02E-31 | 6.18E-30 | Yes | Up |
| SAXN108_0115 | Udp-Phosphate Galactose Phosphotransferase | 0.92 | | 30.39 | 5.09126114 | 3.54E-31 | 7.19E-30 | Yes | Up |
| SAXN108_2286 | Hypothetical Protein | 2.016666667 | | 9.243333333 | 2.395081926 | 4.42E-31 | 8.93E-30 | Yes | Up |
| SAXN108_1463 | Hypothetical Protein | 31.37 | | 3.87 | -2.832195056 | 6.73E-31 | 1.35E-29 | Yes | Down |
| SAXN108_2274 | Hypothetical Protein | 14.08333333 | | 2.813333333 | -2.135407795 | 9.06E-31 | 1.80E-29 | Yes | Down |
| SAXN108_2907 | Arginine Repressor Family Protein | 11.61 | | 54.57333333 | 2.411957953 | 3.03E-30 | 6.00E-29 | Yes | Up |
| SAXN108_0306 | Triacylglycerol Lipase | 3.996666667 | | 24.95333333 | 2.808727927 | 4.92E-29 | 9.66E-28 | Yes | Up |
| SAXN108_2231 | Hypothetical Protein | 129.25 | | 22.15666667 | -2.385293177 | 9.44E-29 | 1.84E-27 | Yes | Down |
| SAXN108_2967 | Collagen Adhesin | 120.36 | | 648.4966667 | 2.606884955 | 4.85E-28 | 9.40E-27 | Yes | Up |
| SAXN108_1513 | Hypothetical Protein | 20.8 | | 65.01333333 | 1.829331153 | 7.51E-28 | 1.45E-26 | Yes | Up |
| SAXN108_0112 | Hypothetical Protein | 35.05333333 | | 109.4 | 1.82940478 | 8.18E-28 | 1.57E-26 | Yes | Up |
| SAXN108_0155 | Hypothetical Protein | 86.04 | | 16.04 | -2.221751072 | 1.02E-27 | 1.94E-26 | Yes | Down |
| SAXN108_2264 | Hypothetical Protein | 68.28666667 | | 9.483333333 | -2.666322962 | 1.22E-27 | 2.30E-26 | Yes | Down |
| SAXN108_2656 | Hypothetical Protein | 476.2933333 | | 74.31 | -2.495065963 | 1.56E-27 | 2.94E-26 | Yes | Down |
| SAXN108_0206 | Staphylocoagulase | 5.473333333 | | 38.71 | 2.996771275 | 2.09E-27 | 3.90E-26 | Yes | Up |
| SAXN108_0965 | Coa-Disulfide Reductase | 219.3733333 | | 499.7566667 | 1.375835779 | 2.16E-27 | 4.00E-26 | Yes | Up |
| SAXN108_2650 | Respiratory Nitrate Reductase Delta Chain | 163.32 | | 24.49333333 | -2.556328414 | 3.53E-27 | 6.50E-26 | Yes | Down |
| SAXN108_2704 | Hypothetical Protein | 226.9733333 | | 57.00666667 | -1.806036049 | 3.63E-27 | 6.65E-26 | Yes | Down |
| SAXN108_2922 | Hypothetical Protein | 2.533333333 | | 12.68666667 | 2.502557948 | 5.37E-27 | 9.78E-26 | Yes | Up |
| SAXN108_0215 | Putative Extracellular Solute-Bindinglipoprotein | 53.6 | | 15.79666667 | -1.567960091 | 1.56E-26 | 2.83E-25 | Yes | Down |
| SAXN108_0122 | Putative Purine Nucleoside Phosphorylase | 18.23333333 | | 2.603333333 | -2.62038912 | 4.25E-26 | 7.64E-25 | Yes | Down |
| SAXN108_0169 | N-Acetyl-Gamma-Glutamyl-Phosphate Reductase | 1.15 | | 7.323333333 | 2.86267436 | 4.76E-26 | 8.51E-25 | Yes | Up |
| SAXN108_2546 | Arac Family Regulatory Protein | 4.893333333 | | 17.57666667 | 2.033153165 | 5.44E-26 | 9.66E-25 | Yes | Up |
| SAXN108_1793 | Repressor Of Toxins Rot | 114.54 | | 659.7 | 2.713136372 | 1.39E-25 | 2.46E-24 | Yes | Up |
| SAXN108_2968 | Sodium:Sulfate Symporter Family Protein | 50.19 | | 136.7933333 | 1.642201218 | 1.41E-25 | 2.48E-24 | Yes | Up |
| SAXN108_2150 | Ribonucleoside-Diphosphate Reductase | 4.576666667 | | 0.706666667 | -2.509757789 | 3.11E-25 | 5.42E-24 | Yes | Down |
| SAXN108_0696 | Na+/H+ Antiporter | 27.71333333 | | 64.98666667 | 1.423507178 | 7.54E-25 | 1.31E-23 | Yes | Up |
| SAXN108_2224 | Phage Protein | 9.996666667 | | 1.34 | -2.711626665 | 1.12E-24 | 1.93E-23 | Yes | Down |
| SAXN108_2257 | Hypothetical Protein | 50.99333333 | | 10.88666667 | -2.047394172 | 1.44E-24 | 2.47E-23 | Yes | Down |
| SAXN108_2230 | Hypothetical Protein | 71.14 | | 16.36 | -1.944477815 | 1.46E-24 | 2.49E-23 | Yes | Down |
| SAXN108_1306 | Aerobic Glycerol-3-Phosphate Dehydrogenase | 258.6233333 | | 96.68 | -1.233397049 | 2.17E-24 | 3.68E-23 | Yes | Down |
| SAXN108_0857 | Staphylocoagulase Precursor | 1.123333333 | | 5.45 | 2.472421893 | 2.39E-24 | 4.02E-23 | Yes | Up |
| SAXN108_2608 | Teicoplanin-Resistance Associated Hth-Type Transcriptional Regulator Tcar | 53.24666667 | | 129.5866667 | 1.470158356 | 3.78E-24 | 6.33E-23 | Yes | Up |
| SAXN108_1596 | Hypothetical Protein | 76.22333333 | | 192.4033333 | 1.508077181 | 4.28E-24 | 7.13E-23 | Yes | Up |
| SAXN108_2460 | Acetolactate Synthase%2C Catabolic | 279.14 | | 73.66666667 | -1.730161044 | 2.48E-23 | 4.11E-22 | Yes | Down |
| SAXN108_2282 | Phage Protein | 6.533333333 | | 1.066666667 | -2.422911561 | 2.75E-23 | 4.54E-22 | Yes | Down |
| SAXN108_2952 | Histidinol Dehydrogenase | 1.893333333 | | 7.423333333 | 2.150387818 | 4.27E-23 | 6.99E-22 | Yes | Up |
| SAXN108_1034 | Ferrichrome Abc Transporter | 6.083333333 | | 18.94 | 1.825132703 | 5.91E-23 | 9.63E-22 | Yes | Up |
| SAXN108_1044 | Cysteine Protease | 20.87333333 | | 104.5766667 | 2.497052535 | 9.26E-23 | 1.50E-21 | Yes | Up |
| SAXN108_0097 | Hth-Type Transcriptional Regulator Sars | 109.2033333 | | 297.7266667 | 1.637306889 | 1.44E-22 | 2.32E-21 | Yes | Up |
| SAXN108_1832 | Hypothetical Protein | 2.626666667 | | 32.62 | 3.85679322 | 2.01E-22 | 3.23E-21 | Yes | Up |
| SAXN108_1761 | Formate--Tetrahydrofolate Ligase | 93.04 | | 30.73666667 | -1.404541012 | 2.07E-22 | 3.30E-21 | Yes | Down |
| SAXN108_2924 | Preprotein Translocase Secy Subunit-Likeprotein | 1.166666667 | | 6.36 | 2.629401485 | 2.69E-22 | 4.27E-21 | Yes | Up |
| SAXN108_2552 | Glycerate Dehydrogenase | 370.26 | | 113.63 | -1.51378561 | 2.99E-22 | 4.72E-21 | Yes | Down |
| SAXN108_2758 | Putative Membrane Protein | 34.66666667 | | 68.06 | 1.164798663 | 1.81E-21 | 2.83E-20 | Yes | Up |
| SAXN108_2591 | Hypothetical Protein | 20.15666667 | | 45.50666667 | 1.362588521 | 2.57E-21 | 4.02E-20 | Yes | Up |
| SAXN108_2652 | Nitrate Reductase Subunit Alpha | 330.6733333 | | 70.14 | -2.043134624 | 3.80E-21 | 5.90E-20 | Yes | Down |
| SAXN108_0823 | Hd Superfamily Hydrolase | 29.84333333 | | 77.02666667 | 1.555791725 | 5.66E-21 | 8.74E-20 | Yes | Up |
| SAXN108_1845 | Serine Protease | 0.45 | | 6.996666667 | 4.20533611 | 6.96E-21 | 1.07E-19 | Yes | Up |
| SAXN108_2651 | Nitrate Reductase Subunit Beta | 274.89 | | 50.31666667 | -2.259276468 | 9.75E-21 | 1.49E-19 | Yes | Down |
| SAXN108_2700 | Putative Glycine Betaine/Carnitine/Cholinetransport System Permease Protein | 33.56 | | 89.31 | 1.604056579 | 1.18E-20 | 1.79E-19 | Yes | Up |
| SAXN108_0170 | Putative Ornithine Aminotransferase Precursor | 1.393333333 | | 9.2 | 2.887348667 | 1.52E-20 | 2.29E-19 | Yes | Up |
| SAXN108_2538 | Urease Alpha Subunit | 30.7 | | 63.80666667 | 1.240774344 | 3.71E-20 | 5.59E-19 | Yes | Up |
| SAXN108_0776 | Hypothetical Protein | 61.01333333 | | 171.51 | 1.674110077 | 5.28E-20 | 7.91E-19 | Yes | Up |
| SAXN108_2323 | Sensor Kinase Protein | 2.11 | | 6.36 | 1.779039112 | 5.96E-20 | 8.88E-19 | Yes | Up |
| SAXN108_2266 | Hypothetical Protein | 17.19 | | 2.443333333 | -2.629231842 | 6.65E-20 | 9.86E-19 | Yes | Down |
| SAXN108_2937 | Putative Capsule Synthesis Protein | 0.056666667 | | 32.78 | 9.516199024 | 6.81E-20 | 1.01E-18 | Yes | Up |
| SAXN108_1171 | Antibacterial Protein | 17.29666667 | | 199.7233333 | 3.702144304 | 7.78E-20 | 1.14E-18 | Yes | Up |
| SAXN108_2144 | Phage Protein | 21.63333333 | | 3.896666667 | -2.285003338 | 7.79E-20 | 1.14E-18 | Yes | Down |
| SAXN108_2718 | Oligopeptide Transport Atp-Binding Protein Oppd | 3.603333333 | | 15.41333333 | 2.276670693 | 1.10E-19 | 1.60E-18 | Yes | Up |
| SAXN108_2278 | Hypothetical Protein | 6.68 | | 1.34 | -2.121404008 | 2.07E-19 | 3.00E-18 | Yes | Down |
| SAXN108_2672 | Cation Efflux Family Protein | 50.16333333 | | 103.4266667 | 1.230073222 | 6.33E-19 | 9.10E-18 | Yes | Up |
| SAXN108_0289 | Formate/Nitrite Transporter Family Protein | 383.3933333 | | 126.4066667 | -1.406205304 | 6.35E-19 | 9.10E-18 | Yes | Down |
| SAXN108_1170 | Hypothetical Protein | 6.763333333 | | 17.51 | 1.56266835 | 7.74E-19 | 1.10E-17 | Yes | Up |
| SAXN108_0775 | Hypothetical Protein | 26.65333333 | | 67.38666667 | 1.51594109 | 9.77E-19 | 1.39E-17 | Yes | Up |
| SAXN108_2941 | Poly-Beta-1%2C6-N-Acetyl-D-Glucosamine Export Protein | 1.69 | | 8.41 | 2.525589549 | 1.35E-18 | 1.90E-17 | Yes | Up |
| SAXN108_2701 | Putative Glycinebetaine/Carnitine/Choline-Binding Lipoprotein Precursor | 13.54333333 | | 37.86333333 | 1.673527841 | 1.46E-18 | 2.06E-17 | Yes | Up |
| SAXN108_2921 | Hypothetical Protein | 3.223333333 | | 13.80666667 | 2.279239025 | 2.10E-18 | 2.94E-17 | Yes | Up |
| SAXN108_2720 | Oligopeptide Transporter Putative Membrane Permease Domain | 1.59 | | 8.71 | 2.63016495 | 2.33E-18 | 3.25E-17 | Yes | Up |
| SAXN108_0831 | Thioredoxin Reductase | 259.4466667 | | 484.4266667 | 1.092077084 | 4.03E-18 | 5.58E-17 | Yes | Up |
| SAXN108_1598 | Hypothetical Protein | 244.3966667 | | 588.1933333 | 1.451293491 | 4.77E-18 | 6.58E-17 | Yes | Up |
| SAXN108_2702 | Putative Glycine Betaine/Carnitine/Cholinetransport System Permease Protein | 6.666666667 | | 20.68333333 | 1.813522551 | 5.45E-18 | 7.48E-17 | Yes | Up |
| SAXN108_0968 | Hypothetical Protein | 1.656666667 | | 5.733333333 | 1.99175389 | 6.55E-18 | 8.96E-17 | Yes | Up |
| SAXN108_1870 | Peptidyl-Prolyl Cis-Trans Isomerase | 266.86 | | 795.1533333 | 1.761580091 | 6.80E-18 | 9.26E-17 | Yes | Up |
| SAXN108_2649 | Respiratory Nitrate Reductase Gamma Chain | 166.7033333 | | 45.80666667 | -1.678584638 | 1.74E-17 | 2.36E-16 | Yes | Down |
| SAXN108_0131 | Putative Nucleotidase | 15.05666667 | | 30.97 | 1.225479061 | 2.13E-17 | 2.87E-16 | Yes | Up |
| SAXN108_0175 | Pts System%2C Glucose-Specific Iic Component/ Pts System%2C Glucose-Specific Iib Component/ Pts System%2C Glucose-Specific Iia Component | 72.41 | | 146.0466667 | 1.197591621 | 3.27E-17 | 4.38E-16 | Yes | Up |
| SAXN108_2653 | Tetrapyrrole (Corrin/Porphyrin) Methylase Familyprotein | 539.2733333 | | 81.82666667 | -2.527784691 | 3.75E-17 | 5.01E-16 | Yes | Down |
| SAXN108_1632 | Hypothetical Protein | 26.62 | | 10.1 | -1.207002512 | 4.17E-17 | 5.55E-16 | Yes | Down |
| SAXN108_2545 | Hypothetical Protein | 4.143333333 | | 13.83333333 | 1.921224272 | 4.40E-17 | 5.83E-16 | Yes | Up |
| SAXN108_0256 | Peptidoglycan Hydrolase | 451.84 | | 86.34 | -2.211475049 | 5.17E-17 | 6.81E-16 | Yes | Down |
| SAXN108_2384 | Putative Non-Heme Iron-Containing Ferritin | 425.1933333 | | 821.5666667 | 1.136371932 | 5.44E-17 | 7.14E-16 | Yes | Up |
| SAXN108_0515 | Putative Exported Protein | 309.19 | | 644.7933333 | 1.241365499 | 7.09E-17 | 9.26E-16 | Yes | Up |
| SAXN108_0859 | Hypothetical Protein | 14.63 | | 42.52333333 | 1.722610687 | 7.74E-17 | 1.01E-15 | Yes | Up |
| SAXN108_2647 | Roxygen Sensor Histidine Kinase Nreb | 374.0666667 | | 105.6533333 | -1.635551347 | 7.78E-17 | 1.01E-15 | Yes | Down |
| SAXN108_2781 | Hypothetical Protein | 6.86 | | 1.036666667 | -2.535001662 | 1.14E-16 | 1.47E-15 | Yes | Down |
| SAXN108_2227 | Hypothetical Protein | 13.89333333 | | 1.883333333 | -2.691229496 | 1.45E-16 | 1.86E-15 | Yes | Down |
| SAXN108_2267 | Hypothetical Protein | 33.11333333 | | 4.033333333 | -2.865521878 | 1.55E-16 | 1.99E-15 | Yes | Down |
| SAXN108_2928 | Hypothetical Protein | 2.436666667 | | 27.94666667 | 3.644748118 | 1.64E-16 | 2.09E-15 | Yes | Up |
| SAXN108_1030 | Hypothetical Protein | 55.43666667 | | 4.723333333 | -3.401479247 | 1.91E-16 | 2.42E-15 | Yes | Down |
| SAXN108_1037 | Acetyltransferase Gnat Family Protein | 14.88 | | 33.39666667 | 1.363607742 | 2.80E-16 | 3.53E-15 | Yes | Up |
| SAXN108_0421 | Hypothetical Protein | 10.56 | | 28.41 | 1.612139824 | 2.81E-16 | 3.53E-15 | Yes | Up |
| SAXN108_0118 | Polysaccharide Biosynthesis Protein | 6.7 | | 18.94333333 | 1.694492042 | 4.27E-16 | 5.34E-15 | Yes | Up |
| SAXN108_1846 | Serine Protease | 0.216666667 | | 5.943333333 | 4.900202566 | 5.65E-16 | 7.04E-15 | Yes | Up |
| SAXN108_2283 | Hypothetical Protein | 16.37666667 | | 2.093333333 | -2.77659454 | 5.85E-16 | 7.26E-15 | Yes | Down |
| SAXN108_0852 | Hypothetical Protein | 4.09 | | 20.69 | 2.506700133 | 6.10E-16 | 7.54E-15 | Yes | Up |
| SAXN108_2155 | Hypothetical Protein | 28.12333333 | | 5.59 | -2.156571226 | 6.97E-16 | 8.58E-15 | Yes | Down |
| SAXN108_2253 | Hypothetical Protein | 7.043333333 | | 1.406666667 | -2.123476374 | 7.06E-16 | 8.66E-15 | Yes | Down |
| SAXN108_2915 | Hypothetical Protein | 103.6266667 | | 217.2466667 | 1.253788304 | 8.02E-16 | 9.79E-15 | Yes | Up |
| SAXN108_2592 | Abc Transporter Atp-Binding Protein | 15.50333333 | | 38.49333333 | 1.498063883 | 9.84E-16 | 1.20E-14 | Yes | Up |
| SAXN108_2168 | Hypothetical Protein | 56.58333333 | | 9.656666667 | -2.378332687 | 1.62E-15 | 1.96E-14 | Yes | Down |
| SAXN108_2759 | Putative Transporter Protein | 48.44 | | 97.89 | 1.203287077 | 1.77E-15 | 2.14E-14 | Yes | Up |
| SAXN108_2655 | Nitrite Reductase Large Subunit | 242.1666667 | | 42.14 | -2.32714311 | 2.41E-15 | 2.89E-14 | Yes | Down |
| SAXN108_2136 | Ammonium Transporter | 10.18333333 | | 1.066666667 | -3.059431901 | 2.42E-15 | 2.89E-14 | Yes | Down |
|  |  | 5137.86 | | 1471.59 | -1.670787053 | 2.91E-15 | 3.47E-14 | Yes | Down |
| SAXN108_1102 | Manganese Transport Protein Mnth | 260.8966667 | | 101.6733333 | -1.168402509 | 3.06E-15 | 3.64E-14 | Yes | Down |
| SAXN108_1826 | Hypothetical Protein | 3.663333333 | | 21.54666667 | 2.716146977 | 3.72E-15 | 4.39E-14 | Yes | Up |
| SAXN108_0123 | Putative Transport System Protein | 23.68666667 | | 8.266666667 | -1.325585523 | 5.18E-15 | 6.10E-14 | Yes | Down |
| SAXN108_2280 | Hypothetical Protein | 18.06666667 | | 3.273333333 | -2.280279357 | 5.28E-15 | 6.19E-14 | Yes | Down |
| SAXN108_2808 | Lead%2C Cadmium%2C Zinc And Mercury Transporting Atpase%3B Copper-Translocating P-Type Atpase | 23.97333333 | | 56.98 | 1.439159607 | 5.57E-15 | 6.50E-14 | Yes | Up |
| SAXN108_2748 | Fibronectin Binding Protein Fnbb | 34.39333333 | | 71.55666667 | 1.236262867 | 6.97E-15 | 8.10E-14 | Yes | Up |
| SAXN108_0617 | Serine-Aspartate Repeat-Containing Protein C | 1.033333333 | | 4.363333333 | 2.256117196 | 9.50E-15 | 1.10E-13 | Yes | Up |
| SAXN108_2221 | Phage Protein | 5.146666667 | | 0.866666667 | -2.379889506 | 1.64E-14 | 1.90E-13 | Yes | Down |
| SAXN108_2281 | Phage Protein | 117 | | 20.89 | -2.306398284 | 1.79E-14 | 2.05E-13 | Yes | Down |
| SAXN108_2875 | Hypothetical Protein | 49.69666667 | | 13.07 | -1.750129508 | 2.20E-14 | 2.51E-13 | Yes | Down |
| SAXN108_2226 | Phage Protein | 13.86333333 | | 1.496666667 | -3.029485731 | 2.23E-14 | 2.54E-13 | Yes | Down |
| SAXN108_1374 | Transcription Antiterminator | 29.13 | | 54.61333333 | 1.09897548 | 2.77E-14 | 3.15E-13 | Yes | Up |
| SAXN108_2154 | Hypothetical Protein | 17.51 | | 3.92 | -1.97778164 | 2.93E-14 | 3.32E-13 | Yes | Down |
| SAXN108_0755 | Transcriptional Regulator Mgra Regulator Of Autolytic Activity | 1264.58 | | 4006.936667 | 1.852873203 | 4.23E-14 | 4.77E-13 | Yes | Up |
| SAXN108_0168 | Putative Arginine Biosynthesis Bifunctional Protein | 1.98 | | 6.203333333 | 1.835114329 | 4.42E-14 | 4.97E-13 | Yes | Up |
| SAXN108_0752 | Undecaprenyl Pyrophosphate Phosphatase | 275.9566667 | | 95.46 | -1.343241015 | 6.91E-14 | 7.74E-13 | Yes | Down |
| SAXN108_2377 | Hypothetical Protein | 79.95 | | 28.31333333 | -1.306464147 | 7.62E-14 | 8.50E-13 | Yes | Down |
| SAXN108_2252 | Phage Protein | 6.843333333 | | 1.826666667 | -1.71940572 | 8.82E-14 | 9.80E-13 | Yes | Down |
| SAXN108_1249 | Putative Cell Wall Hydrolase | 1.936666667 | | 7.953333333 | 2.220346078 | 9.26E-14 | 1.02E-12 | Yes | Up |
| SAXN108_0906 | Hypothetical Protein | 138.29 | | 279.8833333 | 1.195605796 | 1.07E-13 | 1.18E-12 | Yes | Up |
| SAXN108_0954 | Glycerophosphoryl Diester Phosphodiesterase%2C Periplasmic | 4.673333333 | | 14.48 | 1.828999553 | 1.34E-13 | 1.47E-12 | Yes | Up |
| SAXN108_2713 | Hypothetical Protein | 797.0166667 | | 1641.823333 | 1.228013161 | 1.87E-13 | 2.04E-12 | Yes | Up |
| SAXN108_2378 | Putative Peptidase | 70.62333333 | | 23.18 | -1.419895231 | 2.02E-13 | 2.19E-12 | Yes | Down |
| SAXN108_1854 | Epidermin Biosynthesis Protein Epib | 0.75 | | 2.583333333 | 1.968778486 | 2.21E-13 | 2.39E-12 | Yes | Up |
| SAXN108_0473 | Hypothetical Protein | 10.23 | | 69.51333333 | 2.90244301 | 2.81E-13 | 3.03E-12 | Yes | Up |
| SAXN108_2951 | Putative Aminotransferase | 2.416666667 | | 8.323333333 | 1.968886687 | 3.25E-13 | 3.49E-12 | Yes | Up |
| SAXN108_1152 | Hypothetical Protein | 7.876666667 | | 43.67 | 2.641075628 | 3.37E-13 | 3.61E-12 | Yes | Up |
| SAXN108_0100 | Lipoprotein | 3.683333333 | | 17.31 | 2.420688844 | 4.34E-13 | 4.63E-12 | Yes | Up |
| SAXN108_1510 | Hypothetical Protein | 3.063333333 | | 9.326666667 | 1.784140722 | 4.65E-13 | 4.94E-12 | Yes | Up |
| SAXN108_2648 | Hypothetical Protein | 201.4533333 | | 60.14 | -1.576898023 | 5.07E-13 | 5.37E-12 | Yes | Down |
| SAXN108_2291 | Hypothetical Protein | 10.45 | | 26.76333333 | 1.54852172 | 5.69E-13 | 6.00E-12 | Yes | Up |
| SAXN108_2163 | Phage Protein | 12.88 | | 1.87 | -2.61122363 | 6.26E-13 | 6.58E-12 | Yes | Down |
| SAXN108_2717 | Oligopeptide Transport Atp-Binding Protein%2C Putative | 2.606666667 | | 9.35 | 2.049537271 | 6.29E-13 | 6.60E-12 | Yes | Up |
| SAXN108_0575 | Transcriptional Regulator Ctsr | 110.93 | | 205.7666667 | 1.079208349 | 8.60E-13 | 8.95E-12 | Yes | Up |
| SAXN108_0433 | Putative Sodium:Dicarboxylate Symporter Protein | 28.06333333 | | 67.39333333 | 1.453290733 | 9.17E-13 | 9.51E-12 | Yes | Up |
| SAXN108_2716 | Putative Transport Protein | 10.44666667 | | 21.80333333 | 1.254147956 | 1.43E-12 | 1.48E-11 | Yes | Up |
| SAXN108_2703 | Putative Glycine Betaine/Carnitine/Cholinetransport Atp-Binding Protein | 24.42666667 | | 59.16666667 | 1.46488166 | 1.92E-12 | 1.97E-11 | Yes | Up |
| SAXN108_0173 | Putative Thiamine Pyrophosphate Enzyme | 34.79666667 | | 67.18 | 1.134835542 | 2.64E-12 | 2.69E-11 | Yes | Up |
| SAXN108_0202 | Pyruvate Formate-Lyase Activating Enzyme | 175.2833333 | | 364.6166667 | 1.248276053 | 2.68E-12 | 2.71E-11 | Yes | Up |
| SAXN108_2954 | Hypothetical Protein | 0.923333333 | | 4.25 | 2.372753088 | 2.92E-12 | 2.94E-11 | Yes | Up |
| SAXN108_2380 | Hypothetical Protein | 50.82333333 | | 117.6466667 | 1.37669977 | 3.71E-12 | 3.72E-11 | Yes | Up |
| SAXN108_1634 | Biotin Carboxylase | 16.53666667 | | 5.103333333 | -1.506126016 | 3.86E-12 | 3.85E-11 | Yes | Down |
| SAXN108_2172 | Hypothetical Protein | 24.84666667 | | 5.373333333 | -2.042923948 | 4.55E-12 | 4.53E-11 | Yes | Down |
| SAXN108_2800 | Ferrous Iron Transport Protein B | 5.363333333 | | 1.506666667 | -1.647436826 | 4.68E-12 | 4.64E-11 | Yes | Down |
| SAXN108_0733 | Hypothetical Protein | 34.27666667 | | 64.11666667 | 1.099539145 | 6.21E-12 | 6.14E-11 | Yes | Up |
| SAXN108_1935 | Phage Terminase%2C Large Subunit | 1.53 | | 6.233333333 | 2.213536734 | 6.39E-12 | 6.30E-11 | Yes | Up |
| SAXN108_2540 | Urease Accessory Protein Uref | 46.68666667 | | 84.56666667 | 1.036545038 | 7.72E-12 | 7.58E-11 | Yes | Up |
| SAXN108_0870 | Hypothetical Protein | 277.01 | | 584.1533333 | 1.267933692 | 1.06E-11 | 1.04E-10 | Yes | Up |
| SAXN108_0205 | Hypothetical Protein | 20.89 | | 50.61333333 | 1.459255173 | 1.26E-11 | 1.23E-10 | Yes | Up |
| SAXN108_0907 | Hypothetical Protein | 49131.29 | | 89793.34667 | 1.01372804 | 1.40E-11 | 1.36E-10 | Yes | Up |
| SAXN108_1050 | Mannosyl-Glycoprotein Endo-Beta-N-Acetylglucosamidase | 110.37 | | 214.25 | 1.141638537 | 1.83E-11 | 1.78E-10 | Yes | Up |
| SAXN108_2161 | Phage Putative Methyltransferase | 8.496666667 | | 1.823333333 | -2.03628493 | 1.95E-11 | 1.89E-10 | Yes | Down |
| SAXN108_2542 | Urease Accessory Protein Ured | 41.89333333 | | 79.30666667 | 1.102979211 | 2.48E-11 | 2.38E-10 | Yes | Up |
| SAXN108_2324 | Response Regulator Protein | 6.17 | | 16.07666667 | 1.555393182 | 2.70E-11 | 2.59E-10 | Yes | Up |
| SAXN108_2170 | Dna Ligase%2C Phage-Associated | 11.83 | | 3.996666667 | -1.373315273 | 3.08E-11 | 2.95E-10 | Yes | Down |
| SAXN108_1151 | Hypothetical Protein | 3.19 | | 16.03333333 | 2.541329419 | 3.11E-11 | 2.96E-10 | Yes | Up |
| SAXN108_0500 | Putative Cobalamin Synthesis Protein | 4.246666667 | | 0.873333333 | -2.08772849 | 3.11E-11 | 2.96E-10 | Yes | Down |
| SAXN108_0904 | Hypothetical Protein | 36.77 | | 71.52333333 | 1.153278581 | 5.01E-11 | 4.75E-10 | Yes | Up |
| SAXN108_2222 | Phage Metallo-Beta-Lactamase Superfamily Protein | 8.993333333 | | 2.32 | -1.775545581 | 6.04E-11 | 5.69E-10 | Yes | Down |
| SAXN108_2909 | Immunodominant Antigen B | 554.4233333 | | 1107.36 | 1.18767714 | 6.43E-11 | 6.04E-10 | Yes | Up |
| SAXN108_2603 | Hypothetical Protein | 151.12 | | 60.53333333 | -1.140562036 | 9.12E-11 | 8.51E-10 | Yes | Down |
| SAXN108_0234 | Putative Teichoic Acid Biosynthesis Protein | 174.9066667 | | 70.93333333 | -1.115299567 | 9.33E-11 | 8.67E-10 | Yes | Down |
| SAXN108_0759 | Anion Transporter | 25.43666667 | | 58.41333333 | 1.390551964 | 1.09E-10 | 1.01E-09 | Yes | Up |
| SAXN108_0523 | Glutamate Synthase Nadph Small Chain | 15.38666667 | | 5.476666667 | -1.296682388 | 1.21E-10 | 1.11E-09 | Yes | Down |
| SAXN108_0490 | Hypothetical Protein | 5.293333333 | | 9.963333333 | 1.09685948 | 1.76E-10 | 1.61E-09 | Yes | Up |
| SAXN108_1125 | Heme Transporter Isda | 6.38 | | 17.37 | 1.628240082 | 2.04E-10 | 1.86E-09 | Yes | Up |
| SAXN108_1637 | Hypothetical Protein | 16.78333333 | | 5.02 | -1.545963338 | 2.09E-10 | 1.90E-09 | Yes | Down |
| SAXN108_2148 | Ribonucleoside-Diphosphate Reductase Subunit | 7.863333333 | | 1.696666667 | -2.028696062 | 2.28E-10 | 2.07E-09 | Yes | Down |
| SAXN108_2332 | Hypothetical Protein | 60.68333333 | | 17.12 | -1.671427607 | 2.57E-10 | 2.33E-09 | Yes | Down |
| SAXN108_1636 | Hypothetical Protein | 13.25333333 | | 4.273333333 | -1.44858079 | 2.72E-10 | 2.45E-09 | Yes | Down |
| SAXN108_0288 | Hypothetical Protein | 71.84333333 | | 137.2233333 | 1.109227937 | 3.45E-10 | 3.10E-09 | Yes | Up |
| SAXN108_0265 | Putative Secretion Accessory Protein Esab/Yukd | 0.8 | | 26.76333333 | 5.062324095 | 3.46E-10 | 3.10E-09 | Yes | Up |
| SAXN108_0196 | Hypothetical Protein | 13.44 | | 40.65333333 | 1.77944014 | 5.20E-10 | 4.63E-09 | Yes | Up |
| SAXN108_2691 | Hypothetical Protein | 99.86333333 | | 202.9833333 | 1.21525495 | 7.25E-10 | 6.43E-09 | Yes | Up |
| SAXN108_2609 | Hypothetical Protein | 42.72666667 | | 76.96 | 1.038122786 | 7.82E-10 | 6.92E-09 | Yes | Up |
| SAXN108_1841 | Serine Protease | 0.263333333 | | 2.79 | 3.569500629 | 8.91E-10 | 7.85E-09 | Yes | Up |
| SAXN108_2249 | Hypothetical Protein | 32.35666667 | | 8.553333333 | -1.73240007 | 8.94E-10 | 7.85E-09 | Yes | Down |
| SAXN108_1126 | Npqtn Cell Wall Anchored Protein Isdc | 1.453333333 | | 11.02 | 3.118409454 | 9.32E-10 | 8.15E-09 | Yes | Up |
| SAXN108_1930 | Phage Major Capsid Protein | 1.856666667 | | 7.506666667 | 2.192109606 | 1.55E-09 | 1.35E-08 | Yes | Up |
| SAXN108_1991 | Ferritin | 743.5133333 | | 300.18 | -1.122322465 | 1.62E-09 | 1.40E-08 | Yes | Down |
| SAXN108_2733 | Hypothetical Protein | 9864.896667 | | 21798.68 | 1.32891674 | 1.88E-09 | 1.62E-08 | Yes | Up |
| SAXN108_2719 | Oligopeptide Transporter Putative Membrane Permease Domain | 1.12 | | 4.706666667 | 2.262702503 | 1.96E-09 | 1.68E-08 | Yes | Up |
| SAXN108_2929 | Hypothetical Protein | 0.99 | | 6.953333333 | 3.069252254 | 2.52E-09 | 2.16E-08 | Yes | Up |
| SAXN108_0099 | Putative Siderophore Transport System Permease | 3.64 | | 9.483333333 | 1.551957702 | 2.56E-09 | 2.18E-08 | Yes | Up |
| SAXN108_1751 | Putative Soluble Hydrogenase Subunit | 26.3 | | 10.74333333 | -1.098728989 | 2.80E-09 | 2.36E-08 | Yes | Down |
| SAXN108_0679 | Hypothetical Protein | 1902.88 | | 733.0333333 | -1.185220252 | 3.43E-09 | 2.88E-08 | Yes | Down |
| SAXN108_1843 | Serine Protease | 0.216666667 | | 2.906666667 | 3.999718319 | 3.54E-09 | 2.96E-08 | Yes | Up |
| SAXN108_0491 | Hypothetical Protein | 336.3966667 | | 706.34 | 1.234714585 | 4.15E-09 | 3.46E-08 | Yes | Up |
| SAXN108_1597 | Hypothetical Protein | 75.51666667 | | 185.06 | 1.443813119 | 5.83E-09 | 4.81E-08 | Yes | Up |
| SAXN108_1430 | Hypothetical Protein | 207.84 | | 413.7866667 | 1.181923048 | 6.31E-09 | 5.20E-08 | Yes | Up |
| SAXN108_2342 | Single-Stranded Dna-Binding Protein | 51.40666667 | | 18.47333333 | -1.298057609 | 7.27E-09 | 5.96E-08 | Yes | Down |
| SAXN108_2248 | Hypothetical Protein | 49.1 | | 12.66666667 | -1.80025891 | 7.38E-09 | 6.03E-08 | Yes | Down |
| SAXN108_0270 | Hypothetical Protein | 16.05 | | 29.81333333 | 1.071468489 | 7.56E-09 | 6.16E-08 | Yes | Up |
| SAXN108_2331 | Hypothetical Protein | 49.21666667 | | 15.81666667 | -1.476196584 | 8.41E-09 | 6.84E-08 | Yes | Down |
| SAXN108_1124 | Iron-Regulated Heme-Iron Binding Protein | 1.79 | | 4.293333333 | 1.445262528 | 9.86E-09 | 7.97E-08 | Yes | Up |
| SAXN108_1429 | Hypothetical Protein | 95.73666667 | | 213.1133333 | 1.318576691 | 1.10E-08 | 8.85E-08 | Yes | Up |
| SAXN108_0522 | Glutamate Synthase Nadph Large Chain | 23.06 | | 6.38 | -1.66030016 | 1.20E-08 | 9.61E-08 | Yes | Down |
| SAXN108_2271 | Hypothetical Protein | 11.13 | | 2.153333333 | -2.19248257 | 1.24E-08 | 9.94E-08 | Yes | Down |
| SAXN108_2382 | Deoxyribose-Phosphate Aldolase | 63.35 | | 124.36 | 1.16052769 | 1.27E-08 | 1.02E-07 | Yes | Up |
| SAXN108_2897 | Hypothetical Protein | 233.7933333 | | 98.24333333 | -1.101992345 | 1.30E-08 | 1.03E-07 | Yes | Down |
| SAXN108_2381 | Pyrimidine-Nucleoside Phosphorylase | 33.47 | | 62.82333333 | 1.10025707 | 1.41E-08 | 1.12E-07 | Yes | Up |
| SAXN108_2165 | Hypothetical Protein | 22.59666667 | | 3.716666667 | -2.463086587 | 1.59E-08 | 1.26E-07 | Yes | Down |
| SAXN108_2160 | Phage C-5 Cytosine-Specific Dna Methylase | 2.8 | | 0.486666667 | -2.323071375 | 2.16E-08 | 1.71E-07 | Yes | Down |
| SAXN108_0141 | Capsular Polysaccharide Synthesis Enzyme Cap8G | 19.7 | | 8.39 | -1.049592289 | 2.36E-08 | 1.86E-07 | Yes | Down |
| SAXN108_1931 | Phage Protein | 6.35 | | 31.67 | 2.463098509 | 2.36E-08 | 1.86E-07 | Yes | Up |
| SAXN108_0243 | Holin-Like Protein | 36.43333333 | | 13.86 | -1.201028641 | 2.81E-08 | 2.20E-07 | Yes | Down |
| SAXN108_0201 | Formate Acetyltransferase | 192.65 | | 388.6 | 1.20690548 | 3.33E-08 | 2.59E-07 | Yes | Up |
| SAXN108_0221 | Pts System%2C Maltose And Glucose-Specific Iic Component / Pts System%2C Maltose And Lucose-Specific Iib Component | 3.526666667 | | 7.186666667 | 1.219634574 | 3.36E-08 | 2.60E-07 | Yes | Up |
| SAXN108_0269 | Hypothetical Protein | 2.6 | | 18.3 | 2.950920572 | 3.37E-08 | 2.60E-07 | Yes | Up |
| SAXN108_0764 | Hypothetical Protein | 153.6933333 | | 476.33 | 1.80958625 | 3.43E-08 | 2.64E-07 | Yes | Up |
| SAXN108_1344 | Aspartokinase | 3.796666667 | | 1.28 | -1.384825902 | 3.60E-08 | 2.76E-07 | Yes | Down |
| SAXN108_2619 | L-Lactate Permease | 952.7433333 | | 1680.06 | 1.014689568 | 4.02E-08 | 3.07E-07 | Yes | Up |
| SAXN108_1633 | Hypothetical Protein | 9.026666667 | | 3.063333333 | -1.373898626 | 4.05E-08 | 3.08E-07 | Yes | Down |
| SAXN108_2539 | Urease Accessory Protein Uree | 31.00333333 | | 58.99666667 | 1.099912034 | 4.15E-08 | 3.15E-07 | Yes | Up |
| SAXN108_0974 | Phospholipid-Binding Protein | 16.35333333 | | 29.19 | 1.016536916 | 4.47E-08 | 3.38E-07 | Yes | Up |
| SAXN108_2146 | Hypothetical Protein | 29.32 | | 4.476666667 | -2.563637204 | 4.53E-08 | 3.42E-07 | Yes | Down |
| SAXN108_2895 | Sensor Kinase Protein | 30.25 | | 11.79333333 | -1.171653718 | 5.09E-08 | 3.81E-07 | Yes | Down |
| SAXN108_2697 | Hypothetical Protein | 86.22666667 | | 37.11333333 | -1.021868646 | 5.32E-08 | 3.96E-07 | Yes | Down |
| SAXN108_0138 | Capsular Polysaccharide Synthesis Enzyme Cap8D | 20.27666667 | | 8.286666667 | -1.104234792 | 5.54E-08 | 4.11E-07 | Yes | Down |
| SAXN108_0142 | Capsular Polysaccharide Synthesis Enzyme Cap8H | 13.96666667 | | 5.873333333 | -1.068076931 | 6.54E-08 | 4.81E-07 | Yes | Down |
| SAXN108_0139 | Capsular Polysaccharide Synthesis Enzyme | 16.15333333 | | 6.86 | -1.051445782 | 6.96E-08 | 5.11E-07 | Yes | Down |
| SAXN108_0735 | Hypothetical Protein | 6.43 | | 19.90333333 | 1.793156859 | 7.89E-08 | 5.75E-07 | Yes | Up |
| SAXN108_1921 | Hypothetical Protein | 1.583333333 | | 3.186666667 | 1.190859165 | 8.03E-08 | 5.84E-07 | Yes | Up |
| SAXN108_2284 | Phage Amidase | 20.33 | | 7.22 | -1.297684895 | 8.67E-08 | 6.27E-07 | Yes | Down |
| SAXN108_1127 | Heme Transporter Isddef%2C Membrane Component Isdd | 0.766666667 | | 2.59 | 1.951024828 | 8.88E-08 | 6.41E-07 | Yes | Up |
| SAXN108_1844 | Serine Protease | 0.21 | | 2.16 | 3.580761404 | 9.20E-08 | 6.63E-07 | Yes | Up |
| SAXN108_2745 | Transcriptional Regulator Sart | 3.156666667 | | 13.80333333 | 2.343758028 | 1.19E-07 | 8.48E-07 | Yes | Up |
| SAXN108_0140 | Capsular Polysaccharide Synthesis Enzyme Cap8F | 25.86333333 | | 10.57 | -1.11152996 | 1.23E-07 | 8.74E-07 | Yes | Down |
| SAXN108_0492 | Putative Lipoprotein | 1.406666667 | | 4.066666667 | 1.723307345 | 1.45E-07 | 1.03E-06 | Yes | Up |
| SAXN108_0137 | Capsular Polysaccharide Synthesis Enzyme | 19.21666667 | | 7.606666667 | -1.150106031 | 1.57E-07 | 1.11E-06 | Yes | Down |
| SAXN108_2152 | Ribonucleotide Reduction Protein Nrdi | 12.29 | | 2.333333333 | -2.233519569 | 1.74E-07 | 1.22E-06 | Yes | Down |
| SAXN108_1842 | Serine Protease | 0.606666667 | | 3.473333333 | 2.655005343 | 1.74E-07 | 1.22E-06 | Yes | Up |
| SAXN108_2386 | Hypothetical Protein | 93.94333333 | | 206.8266667 | 1.279270909 | 2.15E-07 | 1.49E-06 | Yes | Up |
| SAXN108_2270 | Hypothetical Protein | 5.906666667 | | 0.763333333 | -2.768960344 | 2.21E-07 | 1.53E-06 | Yes | Down |
| SAXN108_2618 | L-Lactate Permease | 798.23 | | 1698.723333 | 1.284723682 | 2.32E-07 | 1.60E-06 | Yes | Up |
| SAXN108_0087 | Hypothetical Protein | 8.136666667 | | 16.07 | 1.15792614 | 2.37E-07 | 1.63E-06 | Yes | Up |
| SAXN108_2157 | Hypothetical Protein | 22.70666667 | | 2.163333333 | -3.255652734 | 2.69E-07 | 1.85E-06 | Yes | Down |
| SAXN108_1136 | Z-Ring-Associated Protein | 63.30333333 | | 25.28666667 | -1.144892916 | 3.08E-07 | 2.11E-06 | Yes | Down |
| SAXN108_0144 | Capsular Polysaccharide Synthesis Enzyme Cap8J | 17.37 | | 6.633333333 | -1.211035776 | 3.17E-07 | 2.16E-06 | Yes | Down |
| SAXN108_1829 | Hypothetical Protein | 0.826666667 | | 4.06 | 2.518296553 | 3.43E-07 | 2.33E-06 | Yes | Up |
| SAXN108_2167 | Hypothetical Protein | 37.22333333 | | 8.913333333 | -1.923350299 | 3.78E-07 | 2.55E-06 | Yes | Down |
| SAXN108_1924 | Phage Protein | 1.14 | | 6.59 | 2.677815803 | 4.25E-07 | 2.86E-06 | Yes | Up |
| SAXN108_1928 | Phage Capsid And Scaffold | 1.106666667 | | 7.323333333 | 2.867243713 | 4.55E-07 | 3.04E-06 | Yes | Up |
| SAXN108_1918 | Phage Protein | 0.516666667 | | 1.713333333 | 1.913192233 | 5.63E-07 | 3.70E-06 | Yes | Up |
| SAXN108_1936 | Phage Protein | 0.5 | | 4.786666667 | 3.464630099 | 6.66E-07 | 4.34E-06 | Yes | Up |
| SAXN108_1934 | Portal Protein%2C Phage Associated | 2.223333333 | | 6.67 | 1.772981641 | 8.30E-07 | 5.39E-06 | Yes | Up |
| SAXN108_0149 | Capsular Polysaccharide Synthesis Enzyme | 27.05 | | 11.53333333 | -1.047414427 | 1.03E-06 | 6.66E-06 | Yes | Down |
| SAXN108_2087 | Phage Replication Initiation Protein | 6.62 | | 20.79333333 | 1.834665997 | 1.15E-06 | 7.39E-06 | Yes | Up |
| SAXN108_2801 | Hypothetical Protein | 23.58333333 | | 2.7 | -2.92477741 | 1.19E-06 | 7.60E-06 | Yes | Down |
| SAXN108_2040 | Membrane Protein | 2.96 | | 19.38 | 2.810115868 | 1.26E-06 | 7.99E-06 | Yes | Up |
| SAXN108_1396 | Oligopeptide Transport System Permease Protein Oppc | 11.9 | | 4.906666667 | -1.086937993 | 1.40E-06 | 8.81E-06 | Yes | Down |
| SAXN108_2873 | Aminotransferase | 129.1266667 | | 41.00333333 | -1.451772735 | 1.51E-06 | 9.52E-06 | Yes | Down |
| SAXN108_0136 | Capsular Polysaccharide Synthesis Enzyme | 34.46 | | 12.42666667 | -1.290659694 | 1.78E-06 | 1.11E-05 | Yes | Down |
| SAXN108_2265 | Hypothetical Protein | 21.16 | | 2.12 | -3.103450189 | 1.83E-06 | 1.14E-05 | Yes | Down |
| SAXN108_2234 | Phage Integrase | 12.76333333 | | 5.143333333 | -1.113126288 | 2.27E-06 | 1.41E-05 | Yes | Down |
| SAXN108_0135 | Capsular Polysaccharide Synthesis Enzyme | 24.72333333 | | 9.076666667 | -1.266972597 | 2.30E-06 | 1.42E-05 | Yes | Down |
|  |  | 0 | | 0 | 6.495138403 | 2.34E-06 | 1.44E-05 | Yes | Up |
| SAXN108_2099 | Phage Antirepressor Protein | 7.353333333 | | 19.28666667 | 1.572692489 | 3.62E-06 | 2.17E-05 | Yes | Up |
| SAXN108_0226 | Pts System%2C Galactitol-Specific Iic Component | 1.98 | | 0.526666667 | -1.723241482 | 3.91E-06 | 2.33E-05 | Yes | Down |
| SAXN108_2950 | Imidazoleglycerol-Phosphate Dehydratase | 2.16 | | 6.9 | 1.841664449 | 4.15E-06 | 2.47E-05 | Yes | Up |
| SAXN108_2240 | Hypothetical Protein | 8.323333333 | | 2.803333333 | -1.387205747 | 4.24E-06 | 2.52E-05 | Yes | Down |
| SAXN108_1509 | Hypothetical Protein | 1.266666667 | | 3.316666667 | 1.564574484 | 4.36E-06 | 2.57E-05 | Yes | Up |
| SAXN108_2752 | Gluconate Permease | 33.46666667 | | 12.57333333 | -1.215741848 | 4.38E-06 | 2.58E-05 | Yes | Down |
| SAXN108_0167 | Putative Amino Acid Kinase | 3.753333333 | | 7.873333333 | 1.24791244 | 4.56E-06 | 2.67E-05 | Yes | Up |
| SAXN108_1437 | Mobile Element Protein | 3.043333333 | | 0.01 | -6.473478324 | 4.94E-06 | 2.87E-05 | Yes | Down |
| SAXN108_2536 | Urease Gamma Subunit | 132.0033333 | | 238.6633333 | 1.011588656 | 5.05E-06 | 2.93E-05 | Yes | Up |
| SAXN108_0147 | Capsular Polysaccharide Synthesis Enzyme | 26.24666667 | | 10.87666667 | -1.090079202 | 5.05E-06 | 2.93E-05 | Yes | Down |
| SAXN108_2898 | Alkaline Phosphatase Iii | 2.866666667 | | 0.796666667 | -1.655510729 | 5.37E-06 | 3.10E-05 | Yes | Down |
| SAXN108_2930 | Hypothetical Protein | 3.026666667 | | 13.49666667 | 2.380060169 | 5.62E-06 | 3.23E-05 | Yes | Up |
| SAXN108_2741 | Hypothetical Protein | 9.02 | | 19.04333333 | 1.270102918 | 5.89E-06 | 3.38E-05 | Yes | Up |
| SAXN108_0863 | Hypothetical Protein | 147.2133333 | | 64.44333333 | -1.027026196 | 7.79E-06 | 4.44E-05 | Yes | Down |
| SAXN108_2279 | Hypothetical Protein | 8.45 | | 2.126666667 | -1.806307692 | 8.58E-06 | 4.88E-05 | Yes | Down |
| SAXN108_1250 | Fmhc Protein Of Femab Family | 1.106666667 | | 2.59 | 1.426731842 | 1.22E-05 | 6.84E-05 | Yes | Up |
| SAXN108_1635 | Biotin Carboxyl Carrier Protein | 24.62333333 | | 8.16 | -1.421295872 | 1.45E-05 | 8.02E-05 | Yes | Down |
| SAXN108_2149 | Ribonucleoside-Diphosphate Reductase 2 Alpha Subunit | 5.533333333 | | 1.543333333 | -1.66127413 | 1.54E-05 | 8.51E-05 | Yes | Down |
| SAXN108_0183 | Putative Glutathione Transporter%2C Permease Component | 0.903333333 | | 2.086666667 | 1.412203463 | 1.54E-05 | 8.51E-05 | Yes | Up |
| SAXN108_1345 | Homoserine Dehydrogenase | 29.84666667 | | 11.06666667 | -1.242624053 | 1.55E-05 | 8.53E-05 | Yes | Down |
| SAXN108_2953 | Atp Phosphoribosyltransferase | 1.15 | | 4.796666667 | 2.218451219 | 1.57E-05 | 8.62E-05 | Yes | Up |
| SAXN108_1346 | Threonine Synthase | 23.76333333 | | 10.31 | -1.021092086 | 1.91E-05 | 0.0001042 | Yes | Down |
| SAXN108_2090 | Recombinational Dna Repair Protein Rect (Prophage Associated) | 12.81666667 | | 28.63333333 | 1.340771332 | 2.24E-05 | 0.0001205 | Yes | Up |
| SAXN108_1913 | Putative Cell Wall Hydrolase | 0.473333333 | | 1.483333333 | 1.82561682 | 2.28E-05 | 0.0001226 | Yes | Up |
| SAXN108_1966 | Phage Antirepressor Protein | 9.45 | | 23.60666667 | 1.509278871 | 2.38E-05 | 0.0001273 | Yes | Up |
| SAXN108_2142 | Hypothetical Protein | 3.876666667 | | 0.61 | -2.479963886 | 2.50E-05 | 0.0001334 | Yes | Down |
| SAXN108_2080 | Phage Protein | 3.503333333 | | 14.00666667 | 2.133889095 | 2.57E-05 | 0.0001371 | Yes | Up |
| SAXN108_2753 | Gluconokinase | 28.06666667 | | 9.456666667 | -1.374670184 | 2.73E-05 | 0.0001448 | Yes | Down |
| SAXN108_1330 | Hypothetical Protein | 0.636666667 | | 18.52333333 | 4.978457421 | 2.93E-05 | 0.0001548 | Yes | Up |
| SAXN108_0969 | Clpb Protein | 86.73 | | 162.4233333 | 1.09362009 | 3.05E-05 | 0.0001603 | Yes | Up |
| SAXN108_2085 | Phage Holliday Junction Resolvase | 2.686666667 | | 8.943333333 | 1.931589589 | 3.29E-05 | 0.0001714 | Yes | Up |
| SAXN108_2235 | Hypothetical Protein | 26.7 | | 11.25 | -1.081039025 | 3.38E-05 | 0.0001754 | Yes | Down |
| SAXN108_1917 | Hypothetical Protein | 0.303333333 | | 1.206666667 | 2.161823064 | 4.15E-05 | 0.0002134 | Yes | Up |
| SAXN108_1926 | Phage Capsid And Scaffold | 2.52 | | 8.28 | 1.857861373 | 4.42E-05 | 0.0002265 | Yes | Up |
| SAXN108_2896 | Response Regulator Protein | 23.29333333 | | 8.463333333 | -1.282413755 | 4.74E-05 | 0.0002423 | Yes | Down |
| SAXN108_2190 | Hypothetical Protein | 4.763333333 | | 0.646666667 | -2.694899522 | 4.96E-05 | 0.0002517 | Yes | Down |
| SAXN108_2207 | Hypothetical Protein | 8.686666667 | | 1.573333333 | -2.286809407 | 5.29E-05 | 0.0002679 | Yes | Down |
| SAXN108_1164 | Hypothetical Protein | 0.113333333 | | 1.41 | 3.848546121 | 5.51E-05 | 0.0002779 | Yes | Up |
| SAXN108_2654 | Assimilatory Nitrite Reductase Small Subunit | 387.0833333 | | 64.15666667 | -2.428356742 | 6.26E-05 | 0.0003127 | Yes | Down |
| SAXN108_2297 | Acetolactate Synthase Large Subunit | 14.88 | | 5.526666667 | -1.23737813 | 6.44E-05 | 0.00032 | Yes | Down |
| SAXN108_1165 | Ornithine Carbamoyltransferase | 0.483333333 | | 1.876666667 | 2.109635664 | 6.97E-05 | 0.0003456 | Yes | Up |
| SAXN108_1937 | Phage Protein | 14.52 | | 28.93333333 | 1.158057439 | 9.23E-05 | 0.0004503 | Yes | Up |
| SAXN108_1962 | Hypothetical Protein | 20.72666667 | | 49.69333333 | 1.416580715 | 9.51E-05 | 0.0004617 | Yes | Up |
| SAXN108_0156 | Hypothetical Protein | 581.9433333 | | 237.22 | -1.112126726 | 0.000101166 | 0.0004887 | Yes | Down |
| SAXN108_1131 | Heme-Degrading Monooxygenase Isdg | 8.963333333 | | 21.31666667 | 1.418208297 | 0.000121289 | 0.0005831 | Yes | Up |
| SAXN108_2091 | Atpase Involved In Dna Repair%2C Phage Associated | 6.646666667 | | 14.45333333 | 1.299957335 | 0.00012554 | 0.0006025 | Yes | Up |
| SAXN108_0197 | Putative Sugar Phosphate Transport Protein | 9.903333333 | | 67.83333333 | 2.962006795 | 0.000127549 | 0.0006112 | Yes | Up |
| SAXN108_1318 | Hypothetical Protein | 2.643333333 | | 9.29 | 1.975971456 | 0.000146522 | 0.0006931 | Yes | Up |
| SAXN108_0120 | Putative Lpxag Surface Protein | 126.98 | | 46.79 | -1.249369529 | 0.000147739 | 0.0006978 | Yes | Down |
| SAXN108_1968 | Hypothetical Protein | 177.8166667 | | 344.5833333 | 1.116922736 | 0.000175737 | 0.0008132 | Yes | Up |
| SAXN108_1328 | Phage Protein | 3.073333333 | | 13.89666667 | 2.30114581 | 0.000209648 | 0.0009581 | Yes | Up |
| SAXN108_2385 | Hypothetical Protein | 41.46 | | 75.60333333 | 1.026966836 | 0.000230218 | 0.0010439 | Yes | Up |
| SAXN108_2092 | Hypothetical Protein | 13.10333333 | | 34.26 | 1.551071378 | 0.000246608 | 0.001115 | Yes | Up |
| SAXN108_2218 | Hypothetical Protein | 3.63 | | 0.73 | -2.14425553 | 0.000271189 | 0.001215 | Yes | Down |
| SAXN108_1933 | Phage Minor Capsid Protein | 1.48 | | 4.336666667 | 1.708456539 | 0.000276327 | 0.0012343 | Yes | Up |
| SAXN108_1853 | Lantibiotic Biosynthesis Protein | 0.603333333 | | 1.66 | 1.657015572 | 0.000288045 | 0.0012828 | Yes | Up |
| SAXN108_0971 | Hypothetical Protein | 0.13 | | 0.656666667 | 2.514683582 | 0.000296044 | 0.0013145 | Yes | Up |
| SAXN108_2212 | Hypothetical Protein | 5.016666667 | | 0.843333333 | -2.413451001 | 0.000327921 | 0.0014388 | Yes | Down |
| SAXN108_1925 | Phage Protein | 0.663333333 | | 4.186666667 | 2.818620745 | 0.00033776 | 0.0014724 | Yes | Up |
| SAXN108_2156 | Phage Protein | 2.076666667 | | 0.436666667 | -2.065861098 | 0.000382723 | 0.0016523 | Yes | Down |
| SAXN108_2744 | Surface Protein G | 0.653333333 | | 4.1 | 2.82494683 | 0.000426251 | 0.0018191 | Yes | Up |
| SAXN108_2084 | Hypothetical Protein Sa Bacteriophages 11%2C Mu50B | 5.99 | | 17.28333333 | 1.644067795 | 0.000431679 | 0.001837 | Yes | Up |
| SAXN108_2083 | Phage Protein | 5.796666667 | | 17.33 | 1.694140556 | 0.00044531 | 0.0018815 | Yes | Up |
| SAXN108_2947 | Hisf Cyclase-Like Protein | 7.346666667 | | 13.08 | 1.01290267 | 0.000469763 | 0.0019763 | Yes | Up |
| SAXN108_2242 | Hypothetical Protein | 5.97 | | 2.416666667 | -1.121301099 | 0.000505465 | 0.0021086 | Yes | Down |
| SAXN108_2166 | Hypothetical Protein | 13.45 | | 3.483333333 | -1.806342173 | 0.000516404 | 0.0021452 | Yes | Down |
| SAXN108_2151 | Hypothetical Protein | 2.2 | | 0.106666667 | -4.221257687 | 0.000611615 | 0.0024987 | Yes | Down |
| SAXN108_2089 | Phage Protein | 2.57 | | 8.086666667 | 1.819929223 | 0.000659982 | 0.0026824 | Yes | Up |
| SAXN108_2241 | Hypothetical Protein | 23.94 | | 8.943333333 | -1.266452259 | 0.000660204 | 0.0026824 | Yes | Down |
| SAXN108_2276 | Hypothetical Protein | 8.56 | | 2.786666667 | -1.444823403 | 0.000738303 | 0.0029793 | Yes | Down |
| SAXN108_2775 | Abc Transporter Atp-Binding Protein | 1.48 | | 0.19 | -2.761174083 | 0.000839679 | 0.0033565 | Yes | Down |
| SAXN108_2147 | Phage Protein | 4.286666667 | | 1.29 | -1.553165613 | 0.000878854 | 0.0034756 | Yes | Down |
| SAXN108_1927 | Phage Head-Tail Adaptor | 2.906666667 | | 9.05 | 1.767056776 | 0.000883484 | 0.0034892 | Yes | Up |
| SAXN108_2820 | Hypothetical Protein | 3.34 | | 1.306666667 | -1.163515695 | 0.000901095 | 0.0035541 | Yes | Down |
| SAXN108_1939 | Transcriptional Activator Rinb%2C Phage Associated | 8.423333333 | | 15.24333333 | 1.02046442 | 0.000934085 | 0.0036793 | Yes | Up |
| SAXN108_0308 | Hypothetical Protein | 4.603333333 | | 0.92 | -2.140801543 | 0.001378025 | 0.0052875 | Yes | Down |
| SAXN108_0275 | Hypothetical Protein | 6.706666667 | | 14.16666667 | 1.250946626 | 0.001604363 | 0.0060851 | Yes | Up |
| SAXN108_1163 | Hypothetical Protein | 0.686666667 | | 2.223333333 | 1.85599294 | 0.001610706 | 0.0061013 | Yes | Up |
| SAXN108_2210 | Hypothetical Protein | 44.06666667 | | 14.42666667 | -1.439203484 | 0.001639674 | 0.0062032 | Yes | Down |
| SAXN108_0268 | Hypothetical Protein | 1.673333333 | | 8.646666667 | 2.546797834 | 0.001754331 | 0.0065949 | Yes | Up |
| SAXN108_1128 | Heme Abc Transporter Substrate-Binding Protein | 0.703333333 | | 2.05 | 1.719875873 | 0.001972287 | 0.0073161 | Yes | Up |
| SAXN108_2305 | 3-Isopropylmalate Dehydratase Small Subunit | 12.57333333 | | 5.123333333 | -1.109312449 | 0.002062548 | 0.0076089 | Yes | Down |
| SAXN108_2246 | Hypothetical Protein | 6.476666667 | | 1.226666667 | -2.205641957 | 0.002073121 | 0.0076289 | Yes | Down |
| SAXN108_1963 | Phage Protein | 21.78333333 | | 40.82333333 | 1.058037248 | 0.002162931 | 0.0078811 | Yes | Up |
| SAXN108_0151 | Heme-Degrading Monooxygenase Isdg | 8 | | 14.77 | 1.056142245 | 0.002269209 | 0.0082481 | Yes | Up |
| SAXN108_2086 | Phage Protein | 3.503333333 | | 19.06 | 2.625209434 | 0.002500138 | 0.0090102 | Yes | Up |
| SAXN108_2088 | Single-Stranded Dna-Binding Protein | 1.94 | | 5.76 | 1.713873688 | 0.002600098 | 0.0093139 | Yes | Up |
| SAXN108_0879 | Toprim Domain Protein | 25.40666667 | | 11.16666667 | -1.013175479 | 0.002729043 | 0.0097171 | Yes | Down |
| SAXN108_0145 | Capsular Polysaccharide Synthesis Enzyme | 4.24 | | 1.79 | -1.058976692 | 0.003649986 | 0.0127213 | Yes | Down |
| SAXN108_1336 | Abc Transporter%2C Atp-Binding Protein | 0.24 | | 0.856666667 | 2.000198268 | 0.003677863 | 0.0127815 | Yes | Up |
| SAXN108_2200 | Hypothetical Protein | 6.79 | | 1.3 | -2.220862822 | 0.003759282 | 0.0130257 | Yes | Down |
| SAXN108_2780 | L-Serine Dehydratase%2C Beta Subunit | 3.243333333 | | 1.203333333 | -1.256813664 | 0.004508185 | 0.0151466 | Yes | Down |
| SAXN108_0444 | Hypothetical Protein | 4.633333333 | | 1.533333333 | -1.421260055 | 0.006002068 | 0.0194559 | Yes | Down |
| SAXN108_1923 | Phage Protein | 2.13 | | 5.116666667 | 1.402193022 | 0.007169203 | 0.0229631 | Yes | Up |
| SAXN108_2162 | Hypothetical Protein | 14.28333333 | | 2.29 | -2.51697219 | 0.008270778 | 0.0261526 | Yes | Down |
| SAXN108_2214 | Phage Protein | 3.37 | | 0.603333333 | -2.285935641 | 0.008359798 | 0.0264059 | Yes | Down |
| SAXN108_2443 | Pts System%2C Lactose-Specific Iibc Components | 1.106666667 | | 0.466666667 | -1.055961563 | 0.00936738 | 0.0291845 | Yes | Down |
| SAXN108_2145 | Hypothetical Protein | 6.603333333 | | 0.77 | -2.921800493 | 0.010226256 | 0.0315995 | Yes | Down |
| SAXN108_2582 | Fosfomycin Resistance Protein Fosb | 0.57 | | 3.033333333 | 2.706007586 | 0.010975583 | 0.0335261 | Yes | Up |
| SAXN108_1940 | Hypothetical Protein Sa Bacteriophages 11%2C Mu50B | 14.00666667 | | 29.5 | 1.16937469 | 0.012640723 | 0.038102 | Yes | Up |
| SAXN108_2211 | Hypothetical Protein | 13.67333333 | | 5.16 | -1.281465599 | 0.013249236 | 0.0394947 | Yes | Down |
| SAXN108_2799 | Hypothetical Protein | 30.16333333 | | 9.056666667 | -1.581071712 | 0.013428467 | 0.0399149 | Yes | Down |
| SAXN108_1288 | Hypothetical Protein | 11.14 | | 4.276666667 | -1.209132098 | 0.013830873 | 0.040922 | Yes | Down |
| SAXN108_0824 | Hypothetical Protein | 1.783333333 | | 3.96 | 1.322500961 | 0.014079645 | 0.0415112 | Yes | Up |
| SAXN108_0501 | Hypothetical Protein | 6.046666667 | | 2.1 | -1.376710055 | 0.015089704 | 0.0440079 | Yes | Down |
| SAXN108_1595 | Mobile Element Protein | 12.71333333 | | 5.37 | -1.061346544 | 0.015592854 | 0.0452086 | Yes | Down |
| SAXN108_1337 | Abc Transporter Permease Protein | 0.403333333 | | 1.07 | 1.644935323 | 0.016209525 | 0.0466319 | Yes | Up |
