# Supplementary material for: Staphylococcal accessory regulator SarA-mediated modulation of autolysis and surface charge enables Staphylococcus aureus to evade vancomycin killing
Source: mSystems. 2026 Feb 9;11(3):e01630-25. doi: 10.1128/msystems.01630-25 (PMC13011385; doi:10.1128/msystems.01630-25)
Supplement: Table S4 — Assessment of binding affinity between ABC-like and vancomycin. [file msystems.01630-25-s0006.docx]

**Table S4. Assessment of binding** **affinity between ABC-like and** **vancomycin.**

| **Ligand** | **Analyte** | **Analyte Conc. (μM)** | **KD (M)** | **ka (1/Ms)** | **kd (1/s)** | **Fit model** | **Affinity** |
| --- | --- | --- | --- | --- | --- | --- | --- |
| ABC-like | Vancomycin | 1.56-100 | 6.05E-04 | 1.08E+2 | 6.52E-02 | 1:1 Binding | 605.1 μM |
