# Supplementary material for: Staphylococcal accessory regulator SarA-mediated modulation of autolysis and surface charge enables Staphylococcus aureus to evade vancomycin killing
Source: mSystems. 2026 Feb 9;11(3):e01630-25. doi: 10.1128/msystems.01630-25 (PMC13011385; doi:10.1128/msystems.01630-25)
Supplement: Table S5 — MICs for vancomycin in different S. aureus strains. [file msystems.01630-25-s0007.docx]

**Table S5. MICs for** **vancomycin in different *S. aureus* strains.**

| Strain | MIC (μg/mL) of vancomycin |
| --- | --- |
| X108 | 8 |
| Δ*sarA* | 2 |
| Mu50 | 8 |
| Δ*sarA*-Mu50 | 2 |
| USA300 LAC | 1 |
| Δ*sarA*-USA300 LAC | 1 |
| NCTC8325 | 1 |
| Δ*sarA*-NCTC8325 | 1 |
